# Left Atrial Appendage Occlusion vs Anticoagulants in Dialysis With Atrial Fibrillation

**DOI:** 10.1001/jamanetworkopen.2025.30990

**Published:** 2025-09-09

**Authors:** Gaurav Dhar, Milind A. Phadnis, Suzanne L. Hunt, Holly E. Du, Vincz Ong, Ninad Khandekar, Theresa I. Shireman, Donald Lynch, Sandeep Randhawa, Abhishek Deshmukh, Srikanth Vallurupalli, Nishank Jain

**Affiliations:** 1 Department of Internal Medicine, Rush University, Chicago, Illinois; 2 Department of Biostatistics and Data Science, University of Kansas School of Medicine, Kansas City; 3 Department of Internal Medicine, University of Arkansas for Medical Sciences, Little Rock; 4 Department of Health Services, Policy and Practice, School of Public Health, Brown University, Providence, Rhode Island; 5 Department of Medicine, University of Cincinnati, Cincinnati, Ohio; 6 Department of Cardiovascular Medicine, Mayo Clinic, Rochester, Minnesota

## Abstract

**Importance:**

Patients with kidney failure (KF) receiving long-term dialysis have increased incidence of atrial fibrillation (AF). Patients with KF and AF have increased risk of stroke, death, and bleeding compared with age-matched cohorts. In KF, the use of oral anticoagulants (OACs) increases hemorrhage risk, offsetting potential benefits and making left atrial appendage occlusion (LAAO) a potentially promising solution for risk reduction in AF.

**Objectives:**

To compare the outcomes and safety of OACs and LAAO for risk reduction of AF in patients with KF.

**Design, Setting, and Participants:**

This retrospective cohort study assessed patients with KF enrolled in the US Renal Data System registry between January 1, 2015, and December 31, 2018, who were diagnosed with AF after initiation of long-term dialysis. On average, patients met inclusion criteria for 22 months. Data analysis was completed in May 2024.

**Exposure:**

Medicare claims were used to identify patients who received OACs and LAAO after AF diagnosis. A propensity-matched cohort was created based on the CHA_2_DS_2_-VASc score and year of treatment.

**Main Outcomes and Measures:**

Death, nonfatal bleeds, and nonfatal strokes.

**Results:**

The final cohort included 2344 patients with KF and a new AF diagnosis (median [IQR] age, 66.0 [57.0-73.0] years; 1341 [57.2%] male). Of these patients, 293 received LAAO and 2051 received OACs. A total of 2194 patients (93.6%) were receiving hemodialysis, with the remaining 150 (6.4%) receiving peritoneal dialysis. In the LAAO group, 79 deaths (27.0%) and 21 nonfatal strokes (7.2%) occurred, and 117 patients (39.9%) experienced 234 bleeding events. In the OAC group, 729 deaths (35.5%) and 163 strokes (8.0%) occurred, and 687 patients (33.5%) experienced 1114 bleeding events. After accounting for correlation between bleeds and death, the joint frailty model showed evidence of a treatment association of LAAO compared with OACs on recurrent bleeding events (hazard ratio [HR], 0.74; 95% CI, 0.56-0.98) and death (HR, 0.47; 95% CI, 0.30-0.72). There was a reduced risk of nonfatal stroke in the LAAO group (HR, 0.55; 95% CI, 0.32-0.94), but this finding was not significant after adjustment (HR, 1.18; 95% CI, 0.76-1.81).

**Conclusions and Relevance:**

In this cohort study, LAAO compared with OACs was associated with improved survival and reduced bleeding risk in patients with KF and AF. These findings suggest that these data should be considered in shared decision-making in consideration of LAAO for patients with KF and AF.

## Key Points

**Question:**

Does percutaneous left atrial appendage occlusion compared with an oral anticoagulant offer reduced risk of bleeding, death, and stroke in patients with kidney failure and nonvalvular atrial fibrillation who are undergoing dialysis?

**Findings:**

In this cohort study, 293 patients with kidney failure and atrial fibrillation who received left atrial appendage occlusion were propensity matched to 2051 patients receiving oral anticoagulants. The study found evidence that left atrial appendage occlusion was associated with a lower risk of recurrent bleeding events and death.

**Meaning:**

The findings of this study suggest that in patients with kidney failure and atrial fibrillation, left atrial appendage occlusion may offer safer thromboembolic risk reduction compared with oral anticoagulants.

## Teaser

This cohort study of patients with kidney failure and atrial fibrillation assesses whether treatment with left atrial appendage occlusion vs oral anticoagulants is associated with reduced risk of all-cause death, time to first stroke, and major bleeding events.

## Introduction

People with kidney failure (KF) receiving long-term dialysis have a 3-fold higher risk of nonvalvular atrial fibrillation (AF) than the general population, with prevalence rates nearing 15% to 28% (vs 1%-2%).^1,2,3^ Comorbid AF contributes to a higher risk of stroke or all-cause death in patients with KF compared with the general population (4.57 vs 0.48 per 100 person-years).^4^ Oral anticoagulants (OACs) are prescribed to reduce the risk of stroke for patients with AF; however, due to the acquired risk of bleeding from uremia, these drugs are known to increase bleeding complications in patients with KF.^5,6,7^ Because of this dilemma, less than one-third of patients with KF and AF are prescribed OACs.^8^

An alternative treatment to reduce stroke risk in AF, a percutaneous left atrial appendage occlusion (LAAO) device, became available internationally in 2009 for the general population and in 2015 in the US. This device is proven to reduce the risk of thromboembolic stroke, all-cause death, and major bleeding because 90% of atrial thrombi are restricted to left atrial appendage in AF.^9^ Five-year outcomes of 2 randomized clinical trials (RCTs) and postmarketing data favored LAAO in the prevention of stroke and death compared with OACs in the general population^9,10^; however, the only RCT comparing these 2 treatment options in patients with KF terminated early due to poor recruitment.^11,12^ Thus, there is a lack of outcome and safety data regarding use of these strategies in treating AF in patients with KF. To address this knowledge gap, we used Medicare claims data to compare the outcomes and safety of OACs compared with LAAO among individuals with KF and AF, with propensity score matching to minimize bias by indication. We hypothesized that patients with KF treated with LAAO, compared with those treated with OACs, would have a reduced risk of all-cause death, time to first stroke, and major bleeding events.

## Methods

### Data Source

The US Renal Data System (USRDS) is a national registry that tracks more than 93% of all adult patients undergoing dialysis from dialysis initiation through kidney transplantation or death and incorporates Medicare claims, the primary payer for long-term dialysis. For every patient initiating long-term dialysis, nephrologists submit a Centers for Medicare & Medicaid Services Form 2728 of demographics and comorbidities to their network, which provides the USRDS with regular updates on dialysis modality, transplant, and death. The registry includes demographic and comorbidity conditions for every patient documented on initiation of dialysis, tracks health updates over time, and records the date and causes of death by extracting patient-level Medicare Parts A, B, and D claims. This registry includes tracking of prescription data (exposure), procedure codes (exposure), and diagnosis codes (outcome) over time at the individual patient level.^13^

### Design and Cohort

We conducted a national cohort study of patients with KF diagnosed with AF after starting long-term dialysis using USRDS data files dated January 1, 2015, through December 31, 2018. After applying exclusions, we identified every patient in the dataset who was continuously eligible for Medicare Parts A, B, and D and had an outpatient diagnosis of AF after starting long-term dialysis treatment. We identified an AF diagnosis after the start of long-term dialysis therapy based on an AF diagnosis code appearing on at least 2 outpatient claims appearing 3 days apart or 1 hospital claim. The index date was defined as the date of AF diagnosis; the latest possible index date was December 31, 2018. Comorbidities were assessed 6 months before index dates. Participants were followed up from index date to death or censorship.

All patients undergoing long-term hemodialysis or peritoneal dialysis during the identification period, including those returning to dialysis after failed transplant, were included. We included any patient who was 18 years or older who had survived 6 months or longer from the first USRDS-recorded service. Continuous eligibility for Medicare Parts A, B, and D during the 6 months before the index date was confirmed, and the new AF diagnosis criterion was applied to identify new diagnoses. Patients were excluded if they were younger than 18 years, missing the date of their first USRDS-recorded service, or prescribed OACs before AF diagnosis. We also excluded those who had LAAO device removal codes in their claims data. Institutional review board approval, including waiver of informed consent, was obtained from the University of Arkansas for Medical Sciences on February 23, 2021. University of Kansas Medical Center was the primary data collection, processing, and analysis site. A data use agreement was signed and approved by the USRDS program director on June 24, 2021. Data were downloaded from the USRDS server on October 21, 2021. Data analysis was completed in May 2024, and this study was approved for publication by the USRDS Coordinating Center before submission. This report follows the Strengthening the Reporting of Observational Studies in Epidemiology (STROBE) reporting guideline for cohort studies.

### Variables

Centers for Medicare & Medicaid Services Form 2728 provided information related to dialysis treatment, and demographics, including age, sex, and race, were captured from patient files in the registry. Data on race were collected because there are racial differences in outcomes of AF in patients with KF.^1,2,3,4^ Comorbidities were collected from Form 2728 and were combined with codes appearing on 2 different days in outpatient claims data or once in hospital claims data 6 months before the index dates of AF diagnoses (eTable 1 in Supplement 1). CHA_2_DS_2_-VaSc (congestive heart failure [1 point], hypertension [1 point], age ≥75 years [2 points], diabetes [1 point], history of stroke or transient ischemic attack [2 points], vascular disease [1 point], age 65-74 years [1 point], female sex category [1 point]) and HAS-BLED (hypertension, kidney or liver disease, stroke history, prior bleeding, unstable international normalized ratio, aged >65 years, and drug or alcohol use) scores were calculated.^14^ To capture concomitant medicines, prescriptions for nonsteroidal anti-inflammatory drugs, antiplatelet drugs, statins, antihypertensive medicines, and proton pump inhibitors were flagged only during the 6-month period before treatment assignment.

### Exposure and Switching

The primary exposure variable was assignment to an OAC prescription or a receipt of LAAO. From a new diagnosis of AF after the dialysis start date for a cohort member, each patient receiving an OAC (warfarin, dabigatran, apixaban, rivaroxaban, or edoxaban) was followed up after appearance of the first prescription following a 6-month window without it. There could be instances of individuals being previously exposed to an OAC before receiving LAAO. There were 4 different patterns of treatments prescribed to the patients: individuals who (1) received OACs throughout the observation window (new AF diagnosis till death or censorship); (2) had no OAC treatment before receiving their first OAC prescription during the observation window; (3) had no OAC treatment before receiving LAAO during the observation window; and (4) started with no OAC treatment and then received OAC treatment for some time before receiving LAAO during the observation window. Thus, we chose treatment as a time-dependent exposure for analyses. This choice allowed us to include individuals who received OACs before their LAAO in the analyses in which the first date of OAC receipt was flagged. Individuals were followed up in the OAC arm until they received their LAAO and then switched to the LAAO arm.

### Outcomes

The primary outcome was all-cause death, assessed from the USRDS, because nephrologists are mandated to submit a Death Notification Form, including time and cause of death, within 45 days of the event. The secondary outcome was time-to-first nonfatal ischemic stroke based on hospital claims (eTable 2 in Supplement 1). Safety outcome was any nonfatal major bleeding (gastrointestinal, brain, and others) during the survival period that required hospitalization (eTable 2 in Supplement 1). Bleeding events within 6 weeks of the device placement were designated periprocedural and excluded.

Right censoring occurred when follow-up time ended for a reason other than death, including discontinuation of dialysis, transplantation of kidney, loss to USRDS follow-up, or reaching the end date of the data (December 31, 2018).

### Statistical Analysis

We generated descriptive statistics comparing LAAO vs OAC groups before and after propensity matching and reported continuous variables using means (SDs). We reported categorical variables using numbers (percentages). To minimize confounding by indication, we performed propensity score matching based on the exact CHA_2_DS_2_-VASc score and the year treatment started, herein referenced as index year (LAAO procedure year or first year prescribed OACs) because these covariates were deemed to be most relevant in driving choice of treatment. A logistic regression model was used to calculate the propensity scores. We used the optimal matching method to match cohort members in a 7:1 ratio for OAC and LAAO groups with the caliper set to 0.10 using the SAS procedure psmatch. Members from the OAC group were selected if their propensity score fell within the caliper region of propensity scores for those in the LAAO group. Finally, we compared matched sets to ensure balance in covariates (ie, a standardized difference for selected covariates for matching to be <10%). Because there could still be residual confounding by the remaining covariates, not used in propensity matching, they were subsequently adjusted in the analytical models.^12^ Rationale and justification for propensity matching^15,16^ are provided in eTable 3 in Supplement 1.

To compare time to death, univariable survival analyses using Kaplan-Meier curves were performed comparing OACs vs LAAO with statistical significance determined using a log rank test. These curves only represent crude survival comparisons between LAAO and OAC because they ignore the time-dependent nature of treatment exposure and the influence of other covariates. For time to death, we considered 2 important factors: adjustment for time-fixed covariates (race, age, sex, years receiving dialysis, and concomitant medicines) and adjustment for 2 time-dependent covariates (treatment exposure and nonfatal stroke); these factors were included in a Cox proportional hazards regression model.^17^ To assess the association of LAAO vs OAC with recurrent bleeds, we fit a γ-frailty model using the SAS procedure phreg. This model accomplishes 2 objectives. First, it considers the possibility that different individuals experience different numbers of bleeding events owing to varying levels of frailty (also called patient-to-patient heterogeneity), leading to potentially correlated within-patient time to bleeds. Second, conditional on the frailty, we have the usual Cox proportional hazards regression framework, allowing us to assess the association of all risk factors using hazard ratios (HRs). Thus, we can compare the LAAO vs OAC outcome after adjusting for other risk factors but accounting for recurrent bleeding events (instead of only considering time-to-first bleed).

The aforementioned models are used to separately model time to death, time to recurrent bleeds, and time to first nonfatal stroke. To overcome this limitation, we fit a more advanced model called the Joint Frailty Model using the R package frailtypack. This model allows joint modeling of bleeds (recurrent events) and death (terminal event) accounting for potential correlation between bleeds and death. As noted by Cook and Lawless,^18^ when a terminal event (such as death) prevents us from observing a recurrent event process (such as bleeds), it is possible that the 2 events are correlated, so joint modeling of the 2 events may be necessary.^19^ Thus, our final model choice accomplished all these objectives simultaneously and assessed whether occurrence of a nonfatal stroke is independently associated with increased risk of death (eTable 4 in Supplement 1). Unadjusted and adjusted HRs and their corresponding 95% CIs were reported. A 2-sided α of .05 was used to determine statistical significance. Cox proportional hazards regression model^17,20^ assumptions were assessed using log-log survival plots for categorical covariates and a plot of Schoenfeld residuals vs time for continuous covariates (eFigures 1-4 in Supplement 1). Analyses were generated with SAS software, version 9.4 for Windows (SAS Institute Inc) and R software, version R.4.3.1 (R Foundation for Statistical Computing).

## Results

### Baseline Characteristics

**Figure 1.**
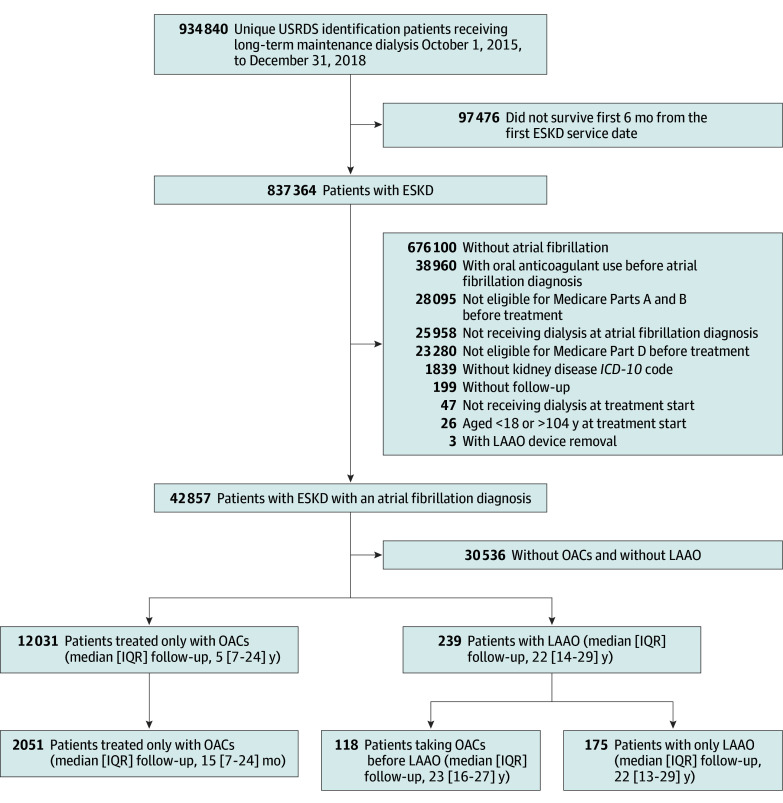
Derivation of the Study Cohort We obtained data from the US Renal Data System (USRDS) on unique patients who received long-term maintenance dialysis (hemodialysis or peritoneal dialysis). Patients in whom kidney transplant failed and who were returning for dialysis were also included. We excluded patients with end-stage kidney disease (ESKD) who did not survive for the first 6 months after starting dialysis or did not have a start date in the system. We further applied inclusion and exclusion criteria to identify patients with ESKD and an atrial fibrillation diagnosis (N = 42 857); 12 031 (28.3%) received only oral anticoagulants (OACs), 293 (0.7%) received left atrial appendage occlusion device (LAAO), and the remaining patients received neither treatment. Of the 293 patients who received LAAO, 175 (59.7%) received LAAO only, whereas the remaining 118 (40.3%) received LAAO after receiving OACs. *ICD-10* indicates *International Statistical Classification of Diseases and Related Health Problems, Tenth Revision.*

After applying exclusions, we successfully matched 293 patients receiving LAAO with 2051 patients receiving OACs based on the CHA_2_DS_2_-VASc score and index year (Table 1). We arrived at a final cohort of 2344 patients with KF and a new AF diagnosis (median [IQR] age, 66 [57.0-73.0] years; 1341 [57.2%] male and 1003 [42.8%] female; 869 [37.1%] were Black or African American, 334 [14.3%] Hispanic White, 1014 [43.3%] non-Hispanic White, and 127 [5.3%] multiracial or other race, including 22 [0.9%] American Indian or Alaska Native, 76 [3.2%] Asian, 25 [1.0%] Native Hawaiian or Pacific Islander and 4 [0.2%] other or multiracial). Within this cohort, 2053 (87.6%) received only OACs, and 293 (12.4%) received LAAO (Figure 1). Of the 293 patients who received LAAO, 175 (59.7%) received LAAO without prior OACs, whereas the remaining 118 (40.3%) received LAAO after receiving OACs (Figure 1).

**Table 1.** Baseline Risk Factors of the Cohort Before and After Propensity Matching^a^

Risk factor	Before propensity score matching			After propensity score matching		
	LAAO (n = 293)	OACs (n = 12 031)	Standardized difference	LAAO (n = 293)	OACs (n = 2051)	Standardized difference
Baseline CHA_2_DS_2_-VASc score, median (IQR)	4 (3-5)	5 (3-6)	−0.30	4 (3-5)	4 (3-5)	0
Demographics						
Age, median (IQR), y	71 (65-77)	69 (60-76)	0.26	71 (65-77)	65 (56-72)	0.58
Sex						
Female	107 (36.5)	5468 (45.4)	−0.18	107 (36.5)	896 (43.7)	−0.15
Male	186 (63.5)	6563 (54.6)		186 (63.5)	1155 (56.3)	
Race						
Black or African American	63 (21.5)	3669 (30.5)	−0.21	63 (21.5)	806 (39.3)	−0.39
Hispanic White	30 (10.2)	1484 (12.3)	−0.07	30 (10.2)	304 (14.8)	−0.14
Non-Hispanic White	185 (63.1)	6188 (51.4)	0.24	185 (63.1)	829 (40.4)	0.47
Other and unknown^b^	15 (5.1)	690 (5.7)	−0.27	15 (5.1)	112 (5.5)	−0.02
Dialysis modality						
Hemodialysis	272 (92.8)	11 024 (91.6)	0.05	272 (92.8)	1922 (93.7)	−0.04
Peritoneal dialysis	21 (7.2)	1007 (8.4)	−0.05	21 (7.2)	129 (6.3)	0.04
Comorbidities						
Stroke	25 (8.5)	1917 (15.9)	−0.23	25 (8.5)	279 (13.6)	−0.16
Diabetes	190 (64.8)	8327 (69.2)	−0.09	190 (64.8)	1208 (58.9)	0.12
Vascular conditions^c^	140 (47.8)	7148 (59.4)	−0.24	140 (47.8)	1126 (54.5)	−0.14
Kidney disease	293 (100.0)	12 031 (100.0)	NA	293 (100.0)	2051 (100)	NA
Baseline HAS-BLED score, median (IQR)^d^	4 (3-4)	4 (3-5)	−0.37	4 (3-4)	4 (3-4)	−0.17
Concomitant medications						
Proton pump inhibitors	93 (31.7)	5117 (42.5)	−0.23	93 (31.7)	909 (44.3)	−0.26
Statins	113 (38.6)	6686 (55.6)	−0.35	113 (38.6)	993 (48.4)	−0.20
Antihypertensives	171 (58.4)	11 373 (94.5)	−0.94	171 (58.4)	1921 (93.7)	−0.91
Apixaban	12 (4.1)	1114 (9.3)	−0.21	12 (4.1)	176 (10.7)	−0.19
NSAIDs or antiplatelet drugs	47 (16.0)	3076 (25.6)	−0.24	47 (16.0)	484 (23.6)	−0.19

Abbreviations: CHA_2_DS_2_-VASc, congestive heart failure [1 point], hypertension [1 point], age ≥75 years [2 points], diabetes [1 point], history of stroke or transient ischemic attack [2 points], vascular disease [1 point], age 65-74 years [1 point], female sex category [1 point]; HAS-BLED, hypertension, kidney or liver disease, stroke history, prior bleeding, unstable international normalized ratio, aged >65 years, and drug or alcohol use; LAAO, left atrial appendage occlusion device; NA, not applicable; NSAIDs, nonsteroidal anti-inflammatory drugs; OACs, oral anticoagulants.

^a^ Data are presented as number (percentage) of patients unless otherwise indicated. Data for sample sizes less than 11 were suppressed per a Data Use Agreement with US Renal Data System.

^b^ Including American Indian or Alaska Native, Asian, Native Hawaiian or Pacific Islander, and other or multiracial.

^c^ Vascular conditions include heart diseases and vascular diseases (peripheral vascular disease, amputations, or any revascularization procedure related to these conditions).

^d^ Due to a lack of data on international normalized ratio, the HAS-BLED score did not include a score for L. Data for alcohol use are not available; antiplatelet drug and NSAID use was included in the baseline HAS-BLED score for D.

A total of 2194 patients (93.6%) were receiving hemodialysis, with the remaining 150 (6.4%) receiving peritoneal dialysis. There were between-group differences in age, sex, race, dialysis modality, time on dialysis, and certain comorbidities (Table 1).

### Outcomes

The median (IQR) follow-up time was 16.1 (8.3-24.7) months. A total of 79 patients (26.9%) in the LAAO group experienced a total of 140 bleeding events. Twenty-six of the 79 patients in the LAAO group experienced a total of 45 bleeding events while receiving OACs before receiving LAAO. A total of 503 patients (24.5%) in the OAC group experienced 762 bleeding events.

### Treatment Associations

**Figure 2.**
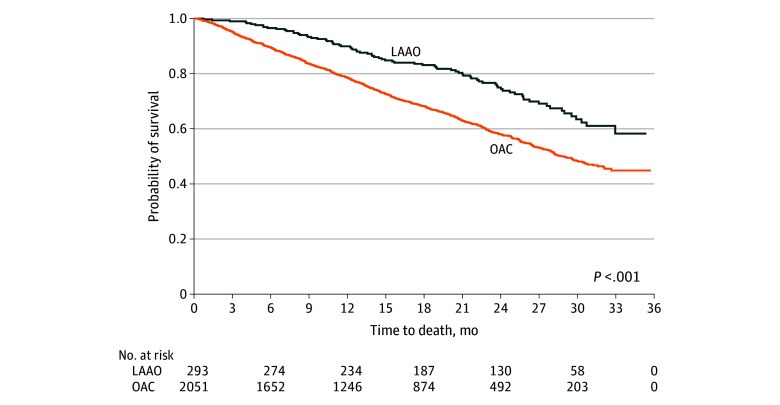
Comparison of Time to Death Between Patients Only Taking Oral Anticoagulant (OACs) vs Those Using the Left Atrial Appendage Occlusion Device (LAAO) in a Univariable Survival Analysis Using Kaplan-Meier Curves The figure shows a comparison of time to death between patients only taking OACs vs those using the LAAO in a univariable survival analysis using Kaplan-Meier curves. Statistical significance was determined using a log-rank test.

Kaplan-Meier analyses demonstrated a reduction in all-cause deaths among patients who ever received LAAO compared with those who ever received OACs (log rank test *P* < .001) (Figure 2). In the joint frailty model, we found a statistically significant positive association between time to recurrent bleeds and time to death (association parameter, 1.56; 95% CI, 1.04-2.08). After accounting for this finding, the joint frailty model showed evidence of a treatment association (LAAO vs OAC) on recurrent bleeding events (HR, 0.74; 95% CI, 0.56-0.98) and consequently a pronounced treatment association (LAAO vs OAC) on death (HR, 0.47; 95% CI, 0.30-0.72) (Table 2). Finally, in univariable analysis, there was reduced risk of nonfatal stroke in the LAAO group vs OAC group (HR, 0.55; 95% CI, 0.32-0.94). However, findings in the full multivariable model were not significant (HR, 1.18; 95% CI, 0.76-1.81).

**Table 2.** Joint Model for Death and Recurrent Bleeding Events^a^

Joint frailty model risk factor	HR (95% CI)	*P* value
**Recurrent bleeding events**		
Treatment		
OACs	1.0 [Reference]	NA
LAAO	0.74 (0.56-0.98)	.03
Race		
Black or African American	1.0 [Reference]	NA
Hispanic White	0.79 (0.63-0.98)	.02
Non-Hispanic White	0.68 (0.58-0.79)	<.001
Other and unknown^b^	0.88 (0.67-1.15)	.38
Sex		
Female	1.0 [Reference]	NA
Male	0.98 (0.85-1.12)	.73
Age group, y		
28-57	1.0 [Reference]	NA
57-66	0.97 (0.80-1.19)	.79
66-73	0.95 (0.77-1.17)	.62
73-95	0.90 (0.73-1.11)	.31
Time receiving dialysis, y		
0-5	1.0 [Reference]	NA
≥5	1.18 (0.98-1.43)	.06
Use of antihypertensives		
No	1.0 [Reference]	NA
Yes	0.70 (0.55-0.89)	.004
Use of statins		
No	1.0 [Reference]	NA
Yes	1.03 (0.88-1.20)	.72
Use of proton pump inhibitor		
No	1.0 [Reference]	NA
Yes	1.45 (1.25-1.69)	<.001
Use of NSAIDs		
No	1.0 [Reference]	NA
Yes	0.97 (0.83-1.14)	.74
No. of prior bleeding events	1.30 (1.19-1.43)	<.001
Patient-to-patient heterogeneity, estimate (95% CI)	0.86 (0.77-0.95)	<.001
Parameter indicating association between recurrent bleeding events and death, estimate (95% CI)	1.56 (1.04-2.08)	<.001
**Death**		
Treatment		
OACs	1.0 [Reference]	NA
LAAO	0.47 (0.30-0.72)	<.001
Stroke		
No	1.0 [Reference]	NA
Yes	1.19 (0.89-1.59)	.22

Abbreviations: HR, hazard ratio; LAAO, left atrial appendage occlusion device; NA, not applicable; NSAIDs, nonsteroidal anti-inflammatory drugs; OACs, oral anticoagulants.

^a^ A joint frailty model was constructed to allow inference on whether patients who are frailer tend to experience more bleeding events, which in turn are positively (or negatively) associated with faster death times. Parameters greater than 1 reflect the association between recurrent bleeding events and death. After accounting for patient-to-patient heterogeneity (frailty >0) and the parameter in this model, inference on all other risk factors (time-fixed or time-dependent covariates) is by means of a Cox proportional hazards regression model.

^b^ Including American Indian or Alaska Native, Asian, Native Hawaiian or Pacific Islander, and other or multiracial.

## Discussion

In this large contemporary cohort of patients with AF and KF, LAAO was associated with a reduced risk of death compared with OACs, likely due to the reduced risk of bleeding. In this population, which is prone to substantial risk of devastating consequences from both stroke and major bleeding,^7,21^ LAAO may offer a safer treatment option than use of OAC prescriptions to reduce the risk of death. To our knowledge, this is the largest study to date comparing these 2 treatment options for patients with AF and KF, a patient population that has been often excluded from RCTs of AF^22,23^ and has been difficult to recruit for RCTs.^24,25^

Contemporary management of AF in patients at a moderate to high risk of stroke consists of OACs in those with acceptable bleeding risk and LAAO device placement in those with contraindications to long-term use of OACs or those with high bleeding risk.^2^ These recommendations are based on RCTs in the population of patients without KF comparing these 2 treatment strategies. In particular, data from PREVAIL^22^ and PROTECT-AF^23^ trials demonstrated that the LAAO device (vs OAC) provided similar reduction in ischemic stroke while significantly reducing risk of hemorrhagic stroke, disabling or fatal stroke, and death.^9,22,23^ A retrospective study^26^ evaluating long-term outcomes of the LAAO device among a subgroup of patients with chronic kidney disease not receiving dialysis demonstrated a 98.6% procedural success rate among these patients. Notably, the subgroup with chronic kidney disease who were not receiving dialysis but underwent LAAO device placement experienced similar risk reductions of stroke and major bleeding as the subgroup without chronic kidney disease.^26,27^ Several cohort studies and registries^28,29^ have reported the safety of LAAO in KF, but none were large enough to compare the efficacy and safety of LAAO with OAC.^30,31^ Most reported safe deployment with acceptable risk of complications (except one^32^) with expected reduction in stroke risk. With the rapid clinical adaptation of the LAAO device in patients with KF and AF and a prior RCT^11,12^ that was limited by poor recruitment of these patients, results from a large prospective registry, such as ours, is an important step forward in providing comparative outcome data on the 2 widely available treatment options.

We observed a reduction in the risk of all-cause death among patients with KF and AF who underwent LAAO placement compared with those receiving OAC prescriptions. These results are in line with a cohort study^33^ in patients with KF that reported 36% increased survival associated with LAAO (n = 92) compared with warfarin prescriptions (n = 114) in this patient population. More recently, a multicenter cohort study^34^ of 220 patients with KF and AF reported reduced risk of death (HR, 0.60; 95% CI, 0.38-0.94) and stroke (HR, 0.19; 95% CI, 0.04-0.96) in patients receiving LAAO compared with OAC prescriptions. However, there were only 30 strokes observed during a median follow up of 5 years. We extend findings of these studies to a larger national cohort of patients with KF and AF. We also observed a higher number of events to generate more precise and more rigorous risk estimates of survival in this patient population even after adjustment for known confounders.

Additionally, we also report that the reduction in death in patients with KF and AF appears to be due to reduction in major bleeding events from the LAAO device compared with OAC prescriptions in this patient population. Consistent with other reports^3,21^ in KF in which bleeding complications increase the risk of death, the current study found a significant association between time to recurrent bleeds and time to death; therefore, the joint model of recurrent bleeds and death allowed us to infer whether patients who experience more bleeds are associated with shorter time to death. In our joint model, the association of LAAO treatment in reducing death for KF with AF became significant compared with OAC prescriptions. This finding suggests that recurrent bleeding events appear to be one of the major drivers of death in this patient population.

### Limitations

Our study has fundamental limitations due to reliance on registry data and bias (despite robust matching) inherent to a retrospective study design. Although there were 184 nonfatal strokes in the cohort, the multivariable Cox proportional hazards regression model did not demonstrate significant differences between LAAO and OAC treatments. Compared with the international cohort studies^34,35^ in patients with KF and AF, bleeding rates are higher in the US cohort probably due to recurrent events measured in this study. Death rates and stroke rates were also higher in the US cohort, suggesting regional differences in event rates for this patient population. Another significant limitation is the lack of a no-treatment arm (ie, no receipt of OACs or LAAO). This was by intention because a significant number of patients are prescribed over-the-counter, low-dose aspirin, which is not documented in the prescription claims. This is supported by the observation in prior studies^33,34^ that even a no-treatment arm had a higher risk of bleeding compared with a LAAO arm. Assessment of bleeding risk using standardized scoring systems, such as HAS-BLED, was performed without international normalized ratio values, which limited granularity of bleeding-related variables. Additionally, dialysis discontinuation and transplant were likely informative censoring events because these events are associated with a patient’s underlying health status and risk of mortality or stroke, which could potentially introduce bias in the results. Despite these limitations, with the rapid clinical adaptation of an LAAO device in this patient population and a prior RCT^11,12^ that were limited by poor recruitment, large prospective registries such as ours can therefore fill the void in evidence until future RCTs conclusively prove the superiority of LAAO over OAC in this patient population.

## Conclusions

In this retrospective cohort study of patients with KF and AF, use of LAAO offered a safer treatment option compared with OACs with significant reduction in risk of bleeding and death. These data should be considered in shared decision-making in consideration of LAAO as a treatment strategy in patients with KF and AF.

## Notes

Supplement 1. **eTable 1.** Codes Used to Define Baseline Characteristics **eTable 2.** Codes Used to Define Outcomes **eTable 3.** Rationale and Justification of Criteria Used for Propensity Matching **eTable 4.** Rationale and Justification of the Joint Frailty Model Over Other Models Including Fine and Gray Model **eFigure 1**. Cox Proportional Hazard Model Assumptions in the Model for Bleeds Were Assessed Using Log-Log Survival Plots for Categorical Covariates **eFigure 2**. Cox Proportional Hazard Model Assumptions in the Model for Death Were Assessed Using Log-Log Survival Plots for Categorical Covariates **eFigure 3**. Cox Proportional Hazard Model Assumptions in the Models for Bleeds Were Assessed Using Plots of Schoenfeld Residuals Versus Time for Continuous Covariates **eFigure 4**. Cox Proportional Hazard Model Assumptions in the Model for Death Were Assessed Using Plots of Schoenfeld Residuals Versus Time for Continuous Covariates

Supplement 2.
Data Sharing Statement
