# Error in Byline

**DOI:** 10.1001/jamanetworkopen.2025.43432

**Published:** 2025-10-20

In the Original Investigation titled “Left Atrial Appendage Occlusion vs Anticoagulants in Dialysis With Atrial Fibrillation,”^1^ published September 9, 2025, Dr Vallurupalli’s name was misspelled in the byline. This article has been corrected.^1^
