# Supplementary material for: Left Atrial Appendage Occlusion vs Anticoagulants in Dialysis With Atrial Fibrillation
Source: JAMA Netw Open. 2025 Sep 9;8(9):e2530990. doi: 10.1001/jamanetworkopen.2025.30990 (PMC12421340; doi:10.1001/jamanetworkopen.2025.30990)
Supplement: Supplement 1. — eTable 1. Codes Used to Define Baseline Characteristics eTable 2. Codes Used to Define Outcomes eTable 3. Rationale and Justification of Criteria Used for Propensity Matching eTable 4. Rationale and Justification of the Joint Frailty Model Over Other Models Including Fine and Gray Model eFigure 1. Cox Proportional Hazard Model Assumptions in the Model for Bleeds Were Assessed Using Log-Log Survival Plots for Categorical Covariates eFigure 2. Cox Proportional Hazard Model Assumptions in the Model for Death Were Assessed Using Log-Log Survival Plots for Categorical Covariates eFigure 3. Cox Proportional Hazard Model Assumptions in the Models for Bleeds Were Assessed Using Plots of Schoenfeld Residuals Versus Time for Continuous Covariates eFigure 4. Cox Proportional Hazard Model Assumptions in the Model for Death Were Assessed Using Plots of Schoenfeld Residuals Versus Time for Continuous Covariates [file jamanetwopen-e2530990-s001.pdf]

Supplemental Online Content

Dhar G, Phadnis MA, Hunt SL, et al. Left atrial appendage occlusion vs anticoagulants in dialysis with atrial fibrillation. *JAMA Netw Open*. 2025;8(9):e2530990. doi:10.1001/jamanetworkopen.2025.30990

eTable 1. Codes Used to Define Baseline Characteristics

eTable 2. Codes Used to Define Outcomes

eTable 3. Rationale and Justification of Criteria Used for Propensity Matching

eTable 4. Rationale and Justification of the Joint Frailty Model Over Other Models Including Fine and Gray Model

eFigure 1. Cox Proportional Hazard Model Assumptions in the Model for Bleeds Were Assessed Using Log-Log Survival Plots for Categorical Covariates

eFigure 2. Cox Proportional Hazard Model Assumptions in the Model for Death Were Assessed Using Log-Log Survival Plots for Categorical Covariates

eFigure 3. Cox Proportional Hazard Model Assumptions in the Models for Bleeds Were Assessed Using Plots of Schoenfeld Residuals Versus Time for Continuous Covariates

eFigure 4. Cox Proportional Hazard Model Assumptions in the Model for Death Were Assessed Using Plots of Schoenfeld Residuals Versus Time for Continuous Covariates

This supplemental material has been provided by the authors to give readers additional information about their work.

© 2025 Dhar G et al. *JAMA Network Open*

eTable 1. Codes Used to Define Baseline Characteristics

Characteristic	Extraction Technique
**Demographics**	
Age	USRenal Data System 'Patients' file
Gender	USRenal Data System 'Patients' file
Race	USRenal Data System 'Patients' file
**Dialysis related factors**	
Treatment modality	USRenal Data System detailed treatment history files
Time on dialysis or dialysis vintage	USRenal Data System 'Patients' file and CMS Form 2728
Etiology of end stage kidney disease	USRenal Data System 'Patients' file
**Comorbidities**	**Codes**
Atrial fibrillation	ICD-10-CM codes. I480-482, I4811, I4819-4821, I4891
Left atrial appendage occlusion device	ICD-10-CM procedure code. 02L73DK
Left atrial appendage occlusion device removal	ICD-10-CM procedure code. 02PA3DZ
CHA_2DS_2-VaSc and HAS-BLED scores	Calculated using diagnosis and procedure codes specified in the Forward-backward mapping schema defined by Webster-Clark 2020, Ref #14
Congestive heart failure	ICD-10-CM codes. I501, I509, I110, I130, I132, I5020-5023, I5082-5084, I5089, I5030-5033, I5040-5043, I50810-50814, 02HA0QZ, 02HA3QZ, 02HA4QZ
Alcohol/Drug use	ICD-10-CM codes. F1010-1011, F1020-1021, F1027, F1096, F1099, F10159, F10180-10182, F10188, F10229, F10231, F10239, F10259, F10280-10282, F10288, F10929, F10950-10951, F10959, F10980, F10982, K700, K709, K7010, K7030, R780, HZ2ZZZZ, HZ30ZZZ, HZ31ZZZ, HZ32ZZZ, HZ33ZZZ, HZ34ZZZ, HZ35ZZZ, HZ36ZZZ, HZ37ZZZ, HZ38ZZZ, HZ39ZZZ,

© 2025 Dhar G et al. *JAMA Network Open*

	HZ3BZZZ, HZ40ZZZ, HZ41ZZZ HZ42ZZZ HZ43ZZZ, HZ44ZZZ, HZ45ZZZ, HZ46ZZZ, HZ47ZZZ, HZ48ZZZ, HZ49ZZZ, HZ4BZZZ, HZ93ZZZ, HZ96ZZZ
Bleeding Predisposition	ICD-10-CM codes. D62, D500-501, D508-513, D518-521, D528-532, D538-539, D550-553, D558-559, D564, D568, D571, D573, D580-582, D588-596, D598-599, D630-631, D638, D640-644, D649, D5700-5702, D5720, D5740, D5780, D6481, D6489, D57211-57212, D57219, D57411-57412, D57419, D57811-57812, D57819, I312, I602, I604, I606-616, I618-619,I621, I629, I6000-6002, I6010-6012, I6030-6032, I6050-6052, I6200-6203, I8501, I8511, K200, K208-210, K226, K228, K250-257, K259-267, K269-277, K279-287, K289, K625, K640-645, K648-649, K661, K920-922, K2900-2901, K2920-2921, K2930-2931, K2940-2941, K2950-2951, K2960-2961, K2970-2971, K2980-2981, K2990-2991, K3182, K5281, K5521, K5700-5701, K5710-5713, K5720-5721, K5730-5733, K5740-5741, K5750-5753, K5780-5781, K5790-5793, M2500, M2508, M25011-25012, M25019, M25021-25022, M25029, M25031-25032, M25039, M25041-25042, M25049, M25051-25052, M25059, M25061-25062, M25069, M25071-25076, N280, N898, N920-921, R040-041, R58, R310-311, R319, R791, R3121, R3129, S064X0A, S064X1A, S064X2A, S064X3A, S064X4A, S064X5A, S064X6A, S064X7A, S064X8A, S064X9A, S065X0A, S065X1A, S065X2A, S065X3A, S065X4A, S065X5A, S065X6A, S065X7A, S065X8A, S065X9A, S066X0A, S066X1A, S066X2A, S066X3A, S066X4A, S066X5A, S066X6A, S066X7A, S066X8A, S066X9A
Diabetes mellitus	ICD-10-CM codes.E108, E109, E118, E119, E138, E139, E1010, E1011, E1021, E1022, E1029, E1036, E1039-1044, E1049, E1051, E1052, E1059, E1065, E1069, E1100, E1101, E1110, E1111, E1121, E1122, E1129, E1136, E1139-1144, E1149, E1151, E1152, E1159, E1165, E1169, E1300, E1301, E1310, E1311, E1321, E1322, E1329, E1336, E1339-1344, E1349, E1351, E1352, E1359, E1365, E1369, E10311, E10319, E103211-103213, E103219-103293, E103299, E10610, E10618, E10620-10622, E10628, E10630, E10638, E10641, E10649, E11311, E11319,E11610, E11618, E11620-11622, E11628, E11630, E11638, E11641, E11649, E13311, E13319, E13610, E13618, E13620-13622, E13628, E13630, E13638, E13641, E13649, E103311-103313, E103319, E103391-103393, E103399, E103411-103413, E103419, E103491-103493, E103499, E103511-103513, E103519, E103521-103523, E103529, E103531-103533, E103539, E103541-103543, E103549, E103551-103553, E103559, E103591-103593, E103599,

© 2025 Dhar G et al. *JAMA Network Open*

	E113211-113213, E113219, E113291-113293, E113299, E113311-113313, E113319, E113391-113393, E113399, E113411-113413, E113419, E113491-113493, E113499, E113511-113513, E113519, E113521-113523, E113529, E113531-113533, E113539, E113541-113543, E113549, E113551-113553, E113559, E113591-113593, E113599, E133211-133213, E133219, E133291-133293, E133299, E133311-133313, E133319, E133391-133393, E133399, E133411-133413, E133419, E133491-133493, E133499, E133511-133513, E133519, E133521-133523, E133529, E133531-133533, E133539, E133541-133543, E133549, E133551-133553, E133559, E133591-133593, E133599, E1037X1, E1037X2, E1037X3, E1037X9, E1137X1, E1137X2, E1137X3, E1137X9, E1337X1, E1337X2, E1337X3, E1337X9
Hypertension	ICD-10-CM codes. I10, I110, I119-120, I129-130, I132, I150-152, I158-161, I169, I973 I1310, I1311, N262
Kidney disease	ICD-10-CM codes. N19, N181-186, N189, N261, N269
Liver disease	ICD-10-CM codes. K700, K702, K709, K730-732, K738-745, K754, K760, K769, K7010-7011, K7030-7031, K7040, K7460, K7469, K7581, K7689
Systemic Embolism/Stroke/TIA	ICD-10-CM codes. G450-452, G458-462, I602, I604, I606-616, I618-619, I636, I638-39, I742-745, I748-749 I2601-2602, I2609, I2690, I2692, I2699, I6000-6002, I6010-6012, I6030-I6032, I6050-6052, I6300, I6302, I6309-6310, I6312, I6319-6320, I6322, I6329-6330, I6339-6340, I6349-6350, I6359, I6789, I7401, I7409-7411, I7419, I63011-I63013, I63019, I63031-63033, I63039,I63111-63113, I63119, I63131-63133, I63139, I63211-63213, I63219, I63231-62233, I63239, I63311-63313, I63319, I63321-63323, I63329, I63331-63333, I63339, I63341-63343, I63349, I63411-63413, I63419, I63421-63423, I63429, I63431-63233, I63441-63443, I63449, I63511-63513, I63519, I63521-I63523, I63529, I63531-63533, I63539, I63541-63543, I63549, I67841, I67848, T800XXA, T81718A, T8172XA, T82817A, T82818A

© 2025 Dhar G et al. *JAMA Network Open*

Peripheral vascular disease	ICD-10-CM codes. A1884, E0851, E0852, E0859, E0865, E0951, E0952, E0959, E1051, E1052, E1059, E1065, E1151, E1152, E1159, E1165, E1351, E1352, E1359, I43, I213, I214, I219-222, I228-229, I420-429, I700, I701, I708, I7025, I731, I739, I791, I798, I2101, I2102, I2109, I2111, I2119, I2121, I2129, I7035, I7045, I7055, I7065, I7075, I7090-7092, I70201-70203, I70208, I70209, I70211-70213, I70218, I70219, I70221-70223, I70228, I70229, I70231-70235, I70238, I70239, I70241-70245, I70248, I70249, I70261-70263, I70268, I70269, I70291-70293, I70298, 70299, I70301-70303, I70308, I70309, I70311-70313, I70318, I70319, I70321-70323, I70328, I70329, I70331-70335, I70338, I70339, I70341-70345, I70348, I70349, I70361-70363, I70368, I70369, I70391-70393, I70398-70399, I70401-70403, I70408, I70409, I70411-70413, I70418, I70419, I70421-70423, I70428, I70429, I70431-70435, I70438, I70439, I70441-70445, I70448, I70449, I70461-70463, I70468, I70469, I70491-70493, I70498, I70499, I70501-70503, I70508, I70509, I70511-70513, I70518, I70519, I70521-70523, I70528, I70529, I70531-70535, I70538, I70539, I70541-70545, I70548, I70549, I70561-70563, I70568, I70569, I70591-70593, I70598, I70599, I70601-70603, I70608, I70609, I70611-70613, I70618, I70619, I70621-70623, I70628, I70629, I70631-70635, I70638, I70639, I70641-70645, I70648, I70649, I70661-70663, I70668, I70669, I70691-70693, I70698-70703, I70708, I70709, I70711-70713, I70718, I70719, I70721-70723, I70728-70729, I70731-70735, I70738, I70739, I70741-70745, I70748, I70749, I70761-70763, I70768, I70769, I70791-70793, I70798, I70799, I21A1, I21A9<br><br>ICD-10 procedure codes. 021W0JG, 021W0JH, 021W0KG, 021W0KH, 027P04Z, 027P0DZ, 027P0ZZ, 027P34Z, 027P3DZ, 027P3ZZ, 027P44Z, 027P4DZ, 027P4ZZ, 027Q04Z, 027Q0DZ, 027Q0ZZ, 027Q34Z, 027Q3DZ, 027Q3ZZ, 027Q44Z, 027Q4DZ, 027Q4ZZ, 027R04Z, 027R0DZ, 027R0ZZ, 027R34Z, 027R3DZ, 027R3ZZ, 027R44Z, 027R4DZ, 027R4ZZ, 027S04Z, 027S0DZ, 027S0ZZ, 027S34Z, 027S3DZ, 027S3ZZ, 027S44Z, 027S4DZ, 027S4ZZ, 027T04Z, 027T0DZ, 027T0ZZ, 027T34Z, 027T3DZ, 027T3ZZ, 027T44Z, 027T4DZ, 027T4ZZ, 027V04Z, 027V0DZ, 027V0ZZ, 027V34Z, 027V3DZ, 027V3ZZ, 027V44Z, 027V4DZ, 027V4ZZ, 027W04Z, 027W0DZ, 027W0ZZ, 027W34Z, 027W3DZ, 027W3ZZ, 027W44Z, 027W4DZ, 027W4ZZ, 027X04Z, 027X0DZ, 027X0ZZ, 027X34Z, 027X3DZ, 027X3ZZ, 027X44Z, 027X4DZ, 027X4ZZ, 02CP0ZZ, 02CP3ZZ, 02CP4ZZ,

	02CQ0ZZ, 02CQ3ZZ, 02CQ4ZZ, 02CR0ZZ, 02CR3ZZ, 02CR4ZZ, 02CS0ZZ, 02CS3ZZ, 02CS4ZZ, 02CT0ZZ, 02CT3ZZ, 02CT4ZZ, 02CV0ZZ, 02CV3ZZ, 02CV4ZZ, 031209B, 031209C, 031209D, 031209F, 031209J, 031209K, 03120AB, 03120AC, 03120AD, 03120AF, 03120AJ, 03120AK, 03120JB, 03120JC, 03120JD, 03120JF, 03120JJ, 03120JK, ,03120KB, 03120KC, 03120KD, 03120KF, 03120KJ, 03120KK, , 03120ZB, 03120ZC, 03120ZD, 03120ZF, 03120ZJ, 03120ZK, 031309B, 031309C, 031309D, 031309F, 031309J, 031309K, 03130AB, 03130AC, 03130AD, 03130AF, 03130AJ, 03130AK,03130JB, 03130JC, 03130JD, 03130JF, 03130JJ, 03130JK, 03130KB, 03130KC, 03130KD, 03130KF, 03130KJ, 03130KK, 03130ZB, 03130ZC, 03130ZD, 03130ZF, 03130ZJ, 03130ZK, 031409B, 031409C, 031409D, 031409F, 031409J, 031409K, 03140AB, 03140AC, 03140AD, 03140AF, 03140AJ, 03140AK, 03140JB, 03140JC, 03140JD, 03140JF, 03140JJ, 03140JK, 03140KB, 03140KC, 03140KD, 03140KF, 03140KJ, 03140KK, 03140ZB, 03140ZC, 03140ZD, 03140ZF, 03140ZJ, 03140ZK, 031509B, 031509C, 031509D, 031509F, 031509J, 031509K, 03150AB, 03150AC, 03150AD, 03150AF, 03150AJ, 03150AK, 03150JB, 03150JC, 03150JD, 03150JF, 03150JJ, 03150JK, 03150KB, 03150KC, 03150KD, 03150KF, 03150KJ, 03150KK, 03150ZB, 03150ZC, 03150ZD, 03150ZF, 03150ZJ, 03150ZK, 031609B, 031609C, 031609D, 031609F, 031609J, 031609K, 03160AB, 03160AC, 03160AD, 03160AF, 03160AJ, 03160AK, 03160JB, 03160JC, 03160JD, 03160JF, 03160JJ, 03160JK, 03160KB, 03160KC, 03160KD, 03160KF, 03160KJ, 03160KK, 03160ZB, 03160ZC, 03160ZD, 03160ZF, 03160ZJ, 03160ZK, 031709D, 031709F, 03170A0, 03170A3, 03170AD, 03170AF, 03170J0, 03170J3, 03170JD, 03170JF, 03170K0, 03170K3, 03170KD, 03170KF, 03170Z0, 03170Z3, 03170ZD, 03170ZF, 031809D, 031809F, 03180A1, 03180A4, 03180AD, 03180AF, 03180J1, 03180J4, 03180JD, 03180JF, 03180K1, 03180K4, 03180KD, 03180KF, 03180Z1, 03180Z4, 03180ZD, 03180ZF, 031909F, 03190A3, 03190AF, 03190J3, 03190JF, 03190K3, 03190KF, 03190Z3, 03190ZF, 031A094, 031A09F, 031A0A4, 031A0AF, 031A0J4, 031A0JF, 031A0K4, 031A0KF, 031A0Z4, 031A0ZF, 031B093, 031B09F, 031B0A3, 031B0AF, 031B0J3, 031B0JF, 031B0K3, 031B0KF<br><br>031B0Z3, 031B0ZF, 031C094, 031C09F, 031C0A4, 031C0AF, 031C0J4, 031C0JF, 031C0K4, 031C0KF, 031C0Z4, 031C0ZF, 031G09G, 031G0AG, 031G0JG, 031G0KG, 031G0ZG, 031H09J, 031H0AJ, 031H0JJ, 031H0KJ, 031H0ZJ, 031J09K, 031J0AK,

	031J0JK, 031J0KK, 031J0ZK, 031K09J, 031K0AJ, 031K0JJ, 031K0KJ, 031K0ZJ, 031L09K, 031L0AK, 031L0JK, 031L0KK, 031L0ZK, 031M09J, 031M0AJ, 031M0JJ, 031M0KJ, 031M0ZJ, 031N09K, 031N0AK, 031N0JK, 031N0KK, 031N0ZK, 037004Z, 037005Z, 037006Z, 037007Z, 03700D6, 03700DZ, 03700EZ, 03700F6, 03700FZ, 03700G6, 03700GZ, 03700Z6, 03700ZZ, 037034Z, 037035Z, 037036Z, 037037Z, 03703D6, 03703DZ, 03703EZ, 03703F6, 03703FZ, 03703G6, 03703GZ, 03703Z6, 03703ZZ, 037044Z, 037045Z, 037046Z, 037047Z, 03704D6, 03704DZ, 03704EZ, 03704F6, 03704FZ, 03704G6, 03704GZ, 03704Z6, 03704ZZ, 037104Z, 037105Z, 037106Z, 037107Z, 03710D6, 03710DZ, 03710EZ, 03710F6, 03710FZ, 03710G6, 03710GZ, 03710Z6, 03710ZZ, 037134Z, 037135Z, 037136Z, 037137Z, 03713D6, 03713DZ, 03713EZ, 03713F6, 03713FZ, 03713G6, 03713GZ, 03713Z6, 03713ZZ, 037144Z, 037145Z, 037146Z, 037147Z, 03714D6, 03714DZ, 03714EZ, 03714F6, 03714FZ, 03714G6, 03714GZ, 03714Z6, 03714ZZ, 037204Z, 037205Z, 037206Z, 037207Z, 03720D6, 03720DZ, 03720EZ, 03720F6, 03720FZ, 03720G6, 03720GZ, 03720Z6, 03720ZZ, 037234Z, 037235Z, 037236Z, 037237Z, 03723D6, 03723DZ, 03723EZ, 03723F6, 03723FZ, 03723G6, 03723GZ, 03723Z6, 03723ZZ, 037244Z, 037245Z, 037246Z, 037247Z, 03724D6, 03724DZ, 03724F6, 03724FZ, 03724G6, 03724GZ, 03724Z6, 03724ZZ, 037304Z, 037305Z, 037306Z, 037307Z, 03730D6, 03730DZ, 03730EZ, 03730F6, 03730FZ, 03730G6, 03730GZ, 03730Z6, 03730ZZ, 037334Z, 037335Z, 037336Z, 037337Z, 03733D6, 03733DZ, 03733EZ, 03733F6, 03733FZ, 03733G6, 03733GZ, 03733Z6, 03733ZZ, 037344Z, 037345Z, 037346Z, 037347Z, 03734D6, 03734DZ, 03734EZ, 03734F6, 03734FZ, 03734G6, 03734GZ, 03734Z6, 03734ZZ, 037404Z, 037405Z, 037406Z, 037407Z, 03740D6, 03740DZ, 03740EZ, 03740F6, 03740FZ, 03740G6, 03740GZ, 03740Z6, 03740ZZ, 037434Z, 037435Z, 037436Z, 037437Z, 03743D6, 03743DZ, 03743EZ, 03743F6, 03743FZ, 03743G6, 03743GZ, 03743Z6, 03743ZZ, 037444Z, 037445Z, 037446Z, 037447Z, 03744D6, 03744DZ, 03744EZ, 03744F6, 03744FZ, 03744G6, 03744GZ, 03744Z6, 03744ZZ, 037504Z, 037505Z, 037506Z, 037507Z, 03750D6, 03750DZ, 03750EZ, 03750F6, 03750FZ, 03750G6, 03750GZ, 03750Z6, 03750ZZ, 037534Z, 037535Z, 037536Z, 037537Z, 03753D6, 03753DZ, 03753EZ, 03753F6, 03753FZ, 03753G6, 03753GZ, 03753Z6, 03753ZZ, 037544Z, 037545Z, 037546Z, 037547Z, 03754D6, 03754DZ, 03754EZ, 03754F6, 03754FZ, 03754G6, 03754GZ, 03754Z6, 03754ZZ, 037604Z, 037605Z,

	037606Z, 037607Z, 03760D6, 03760DZ, 03760EZ, 03760F6, 03760FZ, 03760G6, 03760GZ, 03760Z6, 03760ZZ, 037634Z, 037635Z, 037636Z, 037637Z, 03763D6, 03763DZ, 03763EZ, 03763F6, 03763FZ, 03763G6, 03763GZ, 03763Z6, 03763ZZ, 037644Z, 037645Z, 037646Z, 037647Z, 03764D6, 03764DZ, 03764EZ, 03764F6, 03764FZ, 03764G6, 03764GZ, 03764Z6, 03764ZZ, 037704Z, 037705Z, 037706Z, 037707Z, 03770D6, 03770DZ, 03770EZ, 03770F6, 03770FZ, 03770G6, 03770GZ, 03770Z6, 03770ZZ, 037734Z, 037735Z, 037736Z, 037737Z, 03773D6, 03773DZ, 03773EZ, 03773F6, 03773FZ, 03773G6, 03773GZ, 03773Z6, 03773ZZ, 037744Z, 037745Z, 037746Z, 037747Z, 03774D6, 03774DZ, 03774EZ, 03774F6, 03774FZ, 03774G6, 03774GZ, 03774Z6, 03774ZZ, 037804Z, 037805Z, 037806Z, 037807Z, 03780D6, 03780DZ, 03780EZ, 03780F6, 03780FZ, 03780G6, 03780GZ, 03780Z6, 03780ZZ, 037834Z, 037835Z, 037836Z, 037837Z, 03783D6, 03783DZ, 03783EZ, 03783F6, 03783FZ, 03783G6, 03783GZ, 03783Z6, 03783ZZ, 037844Z, 037845Z, 037846Z, 037847Z, 03784D6, 03784DZ, 03784EZ, 03784F6, 03784FZ, 03784G6, 03784GZ, 03784Z6, 03784ZZ, 037904Z, 037905Z, 037906Z, 037907Z, 03790D6, 03790DZ, 03790EZ, 03790F6, 03790FZ, 03790G6, 03790GZ, 03790Z6, 03790ZZ, 037934Z, 037935Z, 037936Z, 037937Z, 03793D6, 03793DZ, 03793EZ, 03793F6, 03793FZ, 03793G6, 03793GZ, 03793Z6, 03793ZZ, 037944Z, 037945Z, 037946Z, 037947Z, 03794D6, 03794DZ, 03794EZ, 03794F6, 03794FZ, 03794G6, 03794GZ, 03794Z6, 03794ZZ, 037A046, 037A04Z, 037A056, 037A05Z, 037A066, 037A06Z, 037A076, 037A07Z, 037A0D6, 037A0DZ, 037A0E6, 037A0EZ, 037A0F6, 037A0FZ, 037A0G6, 037A0GZ, 037A0Z6, 037A0ZZ, 037A346, 037A34Z, 037A356, 037A35Z, 037A366, 037A36Z, 037A376, 037A37Z, 037A3D6, 037A3DZ, 037A3E6, 037A3EZ, 037A3F6, 037A3FZ, 037A3G6, 037A3GZ, 037A3Z6, 037A3ZZ, 037A446, 037A44Z, 037A456, 037A45Z, 037A466, 037A46Z, 037A476, 037A47Z, 037A4D6, 037A4DZ, 037A4E6, 037A4EZ, 037A4F6, 037A4FZ, 037A4G6, 037A4GZ, 037A4Z6, 037A4ZZ, 037B046, 037B04Z, 037B056, 037B05Z, 037B066, 037B06Z, 037B076, 037B07Z, 037B0D6, 037B0DZ, 037B0E6, 037B0EZ, 037B0F6, 037B0FZ, 037B0G6, 037B0GZ, 037B0Z6, 037B0ZZ, 037B346, 037B34Z, 037B356, 037B35Z, 037B366, 037B36Z, 037B376, 037B37Z, 037B3D6, 037B3DZ, 037B3E6, 037B3EZ, 037B3F6, 037B3FZ, 037B3G6, 037B3GZ, 037B3Z6, 037B3ZZ, 037B446, 037B44Z, 037B456, 037B45Z, 037B466, 037B46Z, 037B476, 037B47Z, 037B4D6, 037B4DZ, 037B4E6, 037B4EZ, 037B4F6, 037B4FZ,

© 2025 Dhar G et al. *JAMA Network Open*

	037B4G6, 037B4GZ, 037B4Z6, 037B4ZZ, 037C046, 037C04Z, 037C056, 037C05Z, 037C066, 037C06Z, 037C076, 037C07Z, 037C0D6, 037C0DZ, 037C0E6, 037C0EZ, 037C0F6, 037C0FZ, 037C0G6, 037C0GZ, 037C0Z6, 037C0ZZ, 037C346, 037C34Z, 037C356, 037C35Z, 037C366, 037C36Z, 037C376, 037C37Z, 037C3D6, 037C3DZ, 037C3E6, 037C3EZ, 037C3F6, 037C3FZ, 037C3G6, 037C3GZ, 037C3Z6, 037C3ZZ, 037C446, 037C44Z, 037C456, 037C45Z, 037C466, 037C46Z, 037C476, 037C47Z, 037C4D6, 037C4DZ, 037C4E6, 037C4EZ, 037C4F6, 037C4FZ, 037C4G6, 037C4GZ, 037C4Z6, 037C4ZZ, 037D046, 037D04Z, 037D056, 037D05Z, 037D066, 037D06Z, 037D076, 037D07Z, 037D0D6, 037D0DZ, 037D0E6, 037D0EZ, 037D0F6, 037D0FZ, 037D0G6, 037D0GZ, 037D0Z6, 037D0ZZ, 037D346, 037D34Z, 037D356, 037D35Z, 037D366, 037D36Z, 037D376, 037D37Z, 037D3D6, 037D3DZ, 037D3E6, 037D3EZ, 037D3F6, 037D3FZ, 037D3G6, 037D3GZ, 037D3Z6, 037D3ZZ, 037D446, 037D44Z, 037D456, 037D45Z, 037D466, 037D46Z, 037D476, 037D47Z, 037D4D6, 037D4DZ, 037D4E6, 037D4EZ, 037D4F6, 037D4FZ, 037D4G6, 037D4GZ, 037D4Z6, 037D4ZZ, 037F046, 037F04Z, 037F056, 037F05Z, 037F066, 037F06Z, 037F076, 037F07Z, 037F0D6, 037F0DZ, 037F0E6, 037F0EZ, 037F0F6, 037F0FZ, 037F0G6, 037F0GZ, 037F0Z6, 037F0ZZ, 037F346, 037F34Z, 037F356, 037F35Z, 037F366, 037F36Z,037F376, 037F37Z, 037F3D6, 037F3DZ, 037F3E6, 037F3EZ, 037F3F6, 037F3FZ, 037F3G6, 037F3GZ, 037F3Z6, 037F3ZZ, 037F446, 037F44Z, 037F456, 037F45Z, 037F466, 037F46Z, 037F476, 037F47Z, 037F4D6, 037F4DZ, 037F4E6, 037F4EZ, 037F4F6, 037F4FZ, 037F4G6, 037F4GZ, 037F4Z6, 037F4ZZ, 037G046, 037G04Z, 037G056, 037G05Z, 037G066, 037G06Z, 037G076, 037G07Z, 037G0D6, 037G0DZ, 037G0E6, 037G0EZ, 037G0F6, 037G0FZ, 037G0G6, 037G0GZ, 037G0Z6, 037G0ZZ, 037H046, 037H04Z, 037H056, 037H05Z, 037H066, 037H06Z, 037H076, 037H07Z, 037H0D6, 037H0DZ, 037H0E6, 037H0EZ, 037H0F6, 037H0FZ, 037H0G6, 037H0GZ, 037H0Z6, 037H0ZZ, 037J046, 037J04Z, 037J056, 037J05Z, 037J066, 037J06Z, 037J076, 037J07Z, 037J0D6, 037J0DZ, 037J0E6, 037J0EZ, 037J0F6, 037J0FZ, 037J0G6, 037J0GZ, 037J0Z6, 037J0ZZ, 037K046, 037K04Z, 037K056, 037K05Z, 037K066, 037K06Z, 037K076, 037K07Z, 037K0D6, 037K0DZ, 037K0E6, 037K0EZ, 037K0F6, 037K0FZ, 037K0G6, 037K0GZ, 037K0Z6, 037K0ZZ, 037L046, 037L04Z, 037L056, 037L05Z, 037L066, 037L06Z, 037L076, 037L07Z, 037L0D6, 037L0DZ, 037L0E6, 037L0EZ, 037L0F6, 037L0FZ, 037L0G6, 037L0GZ, 037L0Z6, 037L0ZZ, 037M046, 037M04Z,

	037M056, 037M05Z, 037M066, 037M06Z, 037M076, 037M07Z, 037M0D6, 037M0DZ, 037M0E6, 037M0EZ, 037M0F6, 037M0FZ, 037M0G6, 037M0GZ, 037M0Z6, 037M0ZZ, 037N046, 037N04Z, 037N056, 037N05Z, 037N066, 037N06Z, 037N076, 037N07Z, 037N0D6, 037N0DZ, 037N0E6, 037N0EZ, 037N0F6, 037N0FZ, 037N0G6, 037N0GZ, 037N0Z6, 037N0ZZ, 037P046, 037P04Z, 037P056, 037P05Z, 037P066, 037P06Z, 037P076, 037P07Z, 037P0D6, 037P0DZ, 037P0E6, 037P0EZ, 037P0F6, 037P0FZ, 037P0G6, 037P0GZ, 037P0Z6, 037P0ZZ, 037Q046, 037Q04Z, 037Q056, 037Q05Z, 037Q066, 037Q06Z, 037Q076, 037Q07Z, 037Q0D6, 037Q0DZ, 037Q0E6, 037Q0EZ, 037Q0F6, 037Q0FZ, 037Q0G6, 037Q0GZ, 037Q0Z6, 037Q0ZZ, 037R046, 037R04Z, 037R056, 037R05Z, 037R066, 037R06Z, 037R076, 037R07Z, 037R0D6, 037R0DZ, 037R0E6, 037R0EZ, 037R0F6, 037R0FZ, 037R0G6, 037R0GZ, 037R0Z6, 037R0ZZ, 037R346, 037R34Z, 037R356, 037R35Z, 037R366, 037R36Z, 037R376, 037R37Z, 037R3D6, 037R3DZ, 037R3E6, 037R3EZ, 037R3F6, 037R3FZ, 037R3G6, 037R3GZ, 037R3Z6, 037R3ZZ, 037R446, 037R44Z, 037R456, 037R45Z, 037R466, 037R46Z, 037R476, 037R47Z, 037R4D6, 037R4DZ, 037R4E6, 037R4EZ, 037R4F6, 037R4FZ, 037R4G6, 037R4GZ, 037R4Z6, 037R4ZZ, 037S046, 037S04Z, 037S056, 037S05Z, 037S066, 037S06Z, 037S076, 037S07Z, 037S0D6, 037S0DZ, 037S0E6, 037S0EZ, 037S0F6, 037S0FZ, 037S0G6, 037S0GZ, 037S0Z6, 037S0ZZ, 037S346, 037S34Z, 037S356, 037S35Z, 037S366, 037S36Z, 037S376, 037S37Z, 037S3D6, 037S3DZ, 037S3E6, 037S3EZ, 037S3F6, 037S3FZ, 037S3G6, 037S3GZ, 037S3Z6, 037S3ZZ, 037S446, 037S44Z, 037S456, 037S45Z, 037S466, 037S46Z, 037S476, 037S47Z, 037S4D6, 037S4DZ, 037S4E6, 037S4EZ, 037S4F6, 037S4FZ, 037S4G6, 037S4GZ, 037S4Z6, 037S4ZZ, 037T046, 037T04Z, 037T056, 037T05Z, 037T066, 037T06Z, 037T076, 037T07Z, 037T0D6, 037T0DZ, 037T0E6, 037T0EZ, 037T0F6, 037T0FZ, 037T0G6, 037T0GZ, 037T0Z6, 037T0ZZ, 037T346, 037T34Z, 037T356, 037T35Z, 037T366, 037T36Z, 037T376, 037T37Z, 037T3D6, 037T3DZ, 037T3E6, 037T3EZ, 037T3F6, 037T3FZ, 037T3G6, 037T3GZ, 037T3Z6, 037T3ZZ, 037T446, 037T44Z, 037T456, 037T45Z, 037T466, 037T46Z, 037T476, 037T47Z, 037T4D6, 037T4DZ, 037T4E6, 037T4EZ, 037T4F6, 037T4FZ, 037T4G6, 037T4GZ, 037T4Z6, 037T4ZZ, 037U046, 037U04Z, 037U056, 037U05Z, 037U066, 037U06Z, 037U076, 037U07Z, 037U0D6, 037U0DZ, 037U0E6, 037U0EZ, 037U0F6, 037U0FZ, 037U0G6, 037U0GZ, 037U0Z6, 037U0ZZ, 037U346, 037U34Z, 037U356, 037U35Z, 037U366, 037U36Z, 037U376, 037U37Z,

© 2025 Dhar G et al. *JAMA Network Open*

	037U3D6, 037U3DZ, 037U3E6, 037U3EZ, 037U3F6, 037U3FZ, 037U3G6, 037U3GZ, 037U3Z6, 037U3ZZ, 037U446, 037U44Z, 037U456, 037U45Z, 037U466, 037U46Z, 037U476, 037U47Z, 037U4D6, 037U4DZ, 037U4E6, 037U4EZ, 037U4F6, 037U4FZ, 037U4G6, 037U4GZ, 037U4Z6, 037U4ZZ, 037V046, 037V04Z, 037V056, 037V05Z, 037V066, 037V06Z, 037V076, 037V07Z, 037V0D6, 037V0DZ, 037V0E6, 037V0EZ, 037V0F6, 037V0FZ, 037V0G6, 037V0GZ, 037V0Z6, 037V0ZZ, 037V346, 037V34Z, 037V356, 037V35Z, 037V366, 037V36Z, 037V376, 037V37Z, 037V3D6, 037V3DZ, 037V3E6, 037V3EZ, 037V3F6, 037V3FZ, 037V3G6, 037V3GZ, 037V3Z6, 037V3ZZ, 037V446, 037V44Z, 037V456, 037V45Z, 037V466, 037V46Z, 037V476, 037V47Z, 037V4D6, 037V4DZ, 037V4E6, 037V4EZ, 037V4F6, 037V4FZ, 037V4G6, 037V4GZ, 037V4Z6, 037V4ZZ, 037Y046, 037Y04Z, 037Y056, 037Y05Z, 037Y066, 037Y06Z, 037Y076, 037Y07Z, 037Y0D6, 037Y0DZ, 037Y0E6, 037Y0EZ, 037Y0F6, 037Y0FZ, 037Y0G6, 037Y0GZ, 037Y0Z6, 037Y0ZZ, 037Y346, 037Y34Z, 037Y356, 037Y35Z, 037Y366, 037Y36Z, 037Y376, 037Y37Z, 037Y3D6, 037Y3DZ, 037Y3E6, 037Y3EZ, 037Y3F6, 037Y3FZ, 037Y3G6, 037Y3GZ, 037Y3Z6, 037Y3ZZ, 037Y446, 037Y44Z, 037Y456, 037Y45Z, 037Y466, 037Y46Z, 037Y476, 037Y47Z, 037Y4D6, 037Y4DZ, 037Y4E6, 037Y4EZ, 037Y4F6, 037Y4FZ, 037Y4G6, 037Y4GZ, 037Y4Z6, 037Y4ZZ, 03C00Z6, 03C00ZZ, 03C03Z6, 03C03ZZ, 03C04Z6, 03C04ZZ, 03C10Z6, 03C10ZZ, 03C13Z6, 03C13ZZ, 03C14Z6, 03C14ZZ, 03C20Z6, 03C20ZZ, 03C23Z6, 03C23ZZ, 03C24Z6, 03C24ZZ, 03C30Z6, 03C30ZZ, 03C33Z6, 03C33ZZ, 03C34Z6, 03C34ZZ, 03C40Z6, 03C40ZZ, 03C43Z6, 03C43ZZ, 03C44Z6, 03C44ZZ, 03C50Z6, 03C50ZZ, 03C53Z6, 03C53ZZ, 03C54Z6, 03C54ZZ, 03C60Z6, 03C60ZZ, 03C63Z6, 03C63ZZ, 03C64Z6, 03C64ZZ, 03C70Z6, 03C70ZZ, 03C73Z6, 03C73ZZ, 03C74Z6, 03C74ZZ, 03C80Z6, 03C80ZZ, 03C83Z6, 03C83ZZ, 03C84Z6, 03C84ZZ, 03C90Z6, 03C90ZZ, 03C93Z6, 03C93ZZ, 03C94Z6, 03C94ZZ, 03CA0Z6, 03CA0ZZ, 03CA3Z6, 03CA3ZZ, 03CA4Z6, 03CA4ZZ, 03CB0Z6, 03CB0ZZ, 03CB3Z6, 03CB3ZZ, 03CB4Z6, 03CB4ZZ, 03CC0Z6, 03CC0ZZ, 03CC3Z6, 03CC3ZZ, 03CC4Z6, 03CC4ZZ, 03CD0Z6, 03CD0ZZ, 03CD3Z6, 03CD3ZZ, 03CD4Z6, 03CD4ZZ, 03CF0Z6, 03CF0ZZ, 03CF3Z6, 03CF3ZZ, 03CF4Z6, 03CF4ZZ, 03CY0Z6, 03CY0ZZ, 03CY3Z6, 03CY3ZZ, 03CY4Z6, 03CY4ZZ, 041009B, 041009C, 041009D, 041009F, 041009G, 041009H, 041009J, 041009K, 041009Q, 041009R, 04100A0, 04100A1, 04100A2, 04100A3, 04100A4, 04100A5, 04100A6, 04100A7, 04100A8, 04100A9, 04100AB, 04100AC, 04100AD, 04100AF, 04100AG, 04100AH,

04100AJ, 04100AK, 04100AQ, 04100AR, 04100J0, 04100J1, 04100J2, 04100J3, 04100J4, 04100J5, 04100J6, 04100J7, 04100J8, 04100J9, 04100JB, 04100JC, 04100JD, 04100JF, 04100JG, 04100JH, 04100JJ, 04100JK, 04100JQ, 04100JR, 04100K0, 04100K1, 04100K2, 04100K3, 04100K4, 04100K5, 04100K6, 04100K7, 04100K8, 04100K9, 04100KB, 04100KC, 04100KD, 04100KF, 04100KG, 04100KH, 04100KJ, 04100KK, 04100KQ, 04100KR, 04100Z0, 04100Z1, 04100Z2, 04100Z3, 04100Z4, 04100Z5, 04100Z6, 04100Z7, 04100Z8, 04100Z9, 04100ZB, 04100ZC, 04100ZD, 04100ZF, 04100ZG, 04100ZH, 04100ZJ, 04100ZK, 04100ZQ, 04100ZR

03120A0-03120A9, **03120J0-03120J9, 03120K0-03120K9**, **03120Z0-03120Z9**, **03130A0-03130A9**, **03130J0-03130J9,** 03130K0-03130K9, 03130Z0-03130Z9, 03140A0-03140A9, 03140J0-03140J9, 03140K0-03140K9, 03140Z0-03140Z9, 03150A0-03150A9, 03150J0-03150J9, 03150K0-03150K9, 03150Z0-03150Z9, 03160A0-03160A9, 03160J0-03160J9, 03160K0-03160K9, 03160Z0-03160Z9, 041049B, 041049C, 041049D, 041049F, 041049G, 041049H, 041049J, 041049K, 041049Q, 041049R, 04104A0, 04104A1, 04104A2, 04104A3, 04104A4, 04104A5, 04104A6, 04104A7, 04104A8, 04104A9, 04104AB, 04104AC, 04104AD, 04104AF, 04104AG, 04104AH, 04104AJ, 04104AK, 04104AQ, 04104AR, 04104J0, 04104J1, 04104J2, 04104J3, 04104J4, 04104J5, 04104J6, 04104J7, 04104J8, 04104J9, 04104JB, 04104JC, 04104JD, 04104JF, 04104JG, 04104JH, 04104JJ, 04104JK, 04104JQ, 04104JR, 04104K0, 04104K1, 04104K2, 04104K3, 04104K4, 04104K5, 04104K6, 04104K7, 04104K8, 04104K9, 04104KB, 04104KC, 04104KD, 04104KF, 04104KG, 04104KH, 04104KJ, 04104KK, 04104KQ, 04104KR, 04104Z0, 04104Z1, 04104Z2, 04104Z3, 04104Z4, 04104Z5, 04104Z6, 04104Z7, 04104Z8, 04104Z9, 04104ZB, 04104ZC, 04104ZD, 04104ZF, 04104ZG, 04104ZH, 04104ZJ, 04104ZK, 04104ZQ, 04104ZR, 04134A3, 04134A4, 04134A5, 04134J3, 04134J4, 04134J5, 04134K3, 04134K4, 04134K5, 04134Z3, 04134Z4, 04134Z5, 04140A3, 04140A4, 04140A5, 04140J3, 04140J4, 04140J5, 04140K3, 04140K4, 04140K5, 04140Z3, 04140Z4, 04140Z5, 04144A3, 04144A4, 04144A5, 04144J3, 04144J4, 04144J5, 04144K3, 04144K4, 04144K5, 04144Z3, 04144Z4, 04144Z5, 041C090, 041C091, 041C092, 041C093, 041C094, 041C095, 041C096, 041C097, 041C098, 041C099, 041C09B, 041C09C, 041C09D, 041C09F, 041C09G,

	041C09H, 041C09J, 041C09K, 041C09Q, 041C09R, 041C0A0, 041C0A1, 041C0A2, 041C0A3, 041C0A4, 041C0A5, 041C0A6, 041C0A7, 041C0A8, 041C0A9, 041C0AB, 041C0AC, 041C0AD, 041C0AF, 041C0AG, 041C0AH, 041C0AJ, 041C0AK, 041C0AQ, 041C0AR, 041C0J0, 041C0J1, 041C0J2, 041C0J3, 041C0J4, 041C0J5, 041C0J6, 041C0J7, 041C0J8, 041C0J9, 041C0JB, 041C0JC, 041C0JD, 041C0JF, 041C0JG, 041C0JH, 041C0JJ, 041C0JK, 041C0JQ, 041C0JR, 041C0K0, 041C0K1, 041C0K2, 041C0K3, 041C0K4, 041C0K5, 041C0K6, 041C0K7, 041C0K8, 041C0K9, 041C0KB, 041C0KC, 041C0KD, 041C0KF, 041C0KG, 041C0KH, 041C0KJ, 041C0KK, 041C0KQ, 041C0KR, 041C0Z0, 041C0Z1, 041C0Z2, 041C0Z3, 041C0Z4, 041C0Z5, 041C0Z6, 041C0Z7, 041C0Z8, 041C0Z9, 041C0ZB, 041C0ZC, 041C0ZD, 041C0ZF, 041C0ZG, 041C0ZH, 041C0ZJ, 041C0ZK, 041C0ZQ, 041C0ZR, 041C490, 041C491, 041C492, 041C493, 041C494, 041C495, 041C496, 041C497, 041C498, 041C499, 041C49B, 041C49C, 041C49D, 041C49F, 041C49G, 041C49H, 041C49J, 041C49K, 041C49Q, 041C49R, 041C4A0, 041C4A1, 041C4A2, 041C4A3, 041C4A4, 041C4A5, 041C4A6, 041C4A7, 041C4A8, 041C4A9, 041C4AB, 041C4AC, 041C4AD, 041C4AF, 041C4AG, 041C4AH, 041C4AJ, 041C4AK, 041C4AQ, 041C4AR, 041C4J0, 041C4J1, 041C4J2, 041C4J3, 041C4J4, 041C4J5, 041C4J6, 041C4J7, 041C4J8, 041C4J9, 041C4JB, 041C4JC, 041C4JD, 041C4JF, 041C4JG, 041C4JH, 041C4JJ, 041C4JK, 041C4JQ, 041C4JR, 041C4K0, 041C4K1, 041C4K2, 041C4K3, 041C4K4, 041C4K5, 041C4K6, 041C4K7, 041C4K8, 041C4K9, 041C4KB, 041C4KC, 041C4KD, 041C4KF, 041C4KG, 041C4KH, 041C4KJ, 041C4KK, 041C4KQ, 041C4KR, 041C4Z0, 041C4Z1, 041C4Z2, 041C4Z3, 041C4Z4, 041C4Z5, 041C4Z6, 041C4Z7, 041C4Z8, 041C4Z9, 041C4ZB, 041C4ZC, 041C4ZD, 041C4ZF, 041C4ZG, 041C4ZH, 041C4ZJ, 041C4ZK, 041C4ZQ, 041C4ZR, 041D090, 041D091, 041D092, 041D093, 041D094, 041D095, 041D096, 041D097, 041D098, 041D099, 041D09B, 041D09C, 041D09D, 041D09F, 041D09G, 041D09H, 041D09J, 041D09K, 041D09Q, 041D09R, 041D0A0, 041D0A1, 041D0A2, 041D0A3, 041D0A4, 041D0A5, 041D0A6, 041D0A7, 041D0A8, 041D0A9, 041D0AB, 041D0AC, 041D0AD, 041D0AF, 041D0AG, 041D0AH, 041D0AJ, 041D0AK, 041D0AQ, 041D0AR, 041D0J0, 041D0J1, 041D0J2, 041D0J3, 041D0J4, 041D0J5, 041D0J6, 041D0J7, 041D0J8, 041D0J9, 041D0JB, 041D0JC, 041D0JD, 041D0JF, 041D0JG, 041D0JH, 041D0JJ, 041D0JK, 041D0JQ, 041D0JR, 041D0K0, 041D0K1, 041D0K2, 041D0K3, 041D0K4, 041D0K5, 041D0K6, 041D0K7, 041D0K8, 041D0K9, 041D0KB, 041D0KC, 041D0KD,

© 2025 Dhar G et al. *JAMA Network Open*

	041D0KF, 041D0KG, 041D0KH, 041D0KJ, 041D0KK, 041D0KQ, 041D0KR, 041D0Z0, 041D0Z1, 041D0Z2, 041D0Z3, 041D0Z4, 041D0Z5, 041D0Z6, 041D0Z7, 041D0Z8, 041D0Z9, 041D0ZB, 041D0ZC, 041D0ZD, 041D0ZF, 041D0ZG, 041D0ZH, 041D0ZJ, 041D0ZK, 041D0ZQ, 041D0ZR, 041D490, 041D491, 041D492, 041D493, 041D494, 041D495, 041D496, 041D497, 041D498, 041D499, 041D49B, 041D49C, 041D49D, 041D49F, 041D49G, 041D49H, 041D49J, 041D49K, 041D49Q, 041D49R, 041D4A0, 041D4A1, 041D4A2, 041D4A3, 041D4A4, 041D4A5, 041D4A6, 041D4A7, 041D4A8, 041D4A9, 041D4AB, 041D4AC, 041D4AD, 041D4AF, 041D4AG, 041D4AH, 041D4AJ, 041D4AK, 041D4AQ, 041D4AR, 041D4J0, 041D4J1, 041D4J2, 041D4J3, 041D4J4, 041D4J5, 041D4J6, 041D4J7, 041D4J8, 041D4J9, 041D4JB, 041D4JC, 041D4JD, 041D4JF, 041D4JG, 041D4JH, 041D4JJ, 041D4JK, 041D4JQ, 041D4JR, 041D4K0, 041D4K1, 041D4K2, 041D4K3, 041D4K4, 041D4K5, 041D4K6, 041D4K7, 041D4K8, 041D4K9, 041D4KB, 041D4KC, 041D4KD, 041D4KF, 041D4KG, 041D4KH, 041D4KJ, 041D4KK, 041D4KQ, 041D4KR, 041D4Z0, 041D4Z1, 041D4Z2, 041D4Z3, 041D4Z4, 041D4Z5, 041D4Z6, 041D4Z7, 041D4Z8, 041D4Z9, 041D4ZB, 041D4ZC, 041D4ZD, 041D4ZF, 041D4ZG, 041D4ZH, 041D4ZJ, 041D4ZK, 041D4ZQ, 041D4ZR, 041E09B, 041E09C, 041E09D, 041E09F, 041E09G, 041E09H, 041E09J, 041E09K, 041E09P, 041E09Q, 041E0A9, 041E0AB, 041E0AC, 041E0AD, 041E0AF, 041E0AG, 041E0AH, 041E0AJ, 041E0AK, 041E0AP, 041E0AQ, 041E0J9, 041E0JB, 041E0JC, 041E0JD, 041E0JF, 041E0JG, 041E0JH, 041E0JJ, 041E0JK, 041E0JP, 041E0JQ, 041E0K9, 041E0KB, 041E0KC, 041E0KD, 041E0KF, 041E0KG, 041E0KH, 041E0KJ, 041E0KK, 041E0KP, 041E0KQ, 041E0Z9, 041E0ZB, 041E0ZC, 041E0ZD, 041E0ZF, 041E0ZG, 041E0ZH, 041E0ZJ, 041E0ZK, 041E0ZP, 041E0ZQ, 041E499, 041E49B, 041E49C, 041E49D, 041E49F, 041E49G, 041E49H, 041E49J, 041E49K, 041E49P, 041E49Q, 041E4A9, 041E4AB, 041E4AC, 041E4AD, 041E4AF, 041E4AG, 041E4AH, 041E4AJ, 041E4AK, 041E4AP, 041E4AQ, 041E4J9, 041E4JB, 041E4JC, 041E4JD, 041E4JF, 041E4JG, 041E4JH, 041E4JJ, 041E4JK, 041E4JP, 041E4JQ, 041E4K9, 041E4KB, 041E4KC, 041E4KD, 041E4KF, 041E4KG, 041E4KH, 041E4KJ, 041E4KK, 041E4KP, 041E4KQ, 041E4Z9, 041E4ZB, 041E4ZC, 041E4ZD, 041E4ZF, 041E4ZG, 041E4ZH, 041E4ZJ, 041E4ZK, 041E4ZP, 041E4ZQ, 041F099, 041F09B, 041F09C, 041F09D, 041F09F, 041F09G, 041F09H, 041F09J, 041F09K, 041F09P, 041F09Q, 041F0A9, 041F0AB, 041F0AC, 041F0AD, 041F0AF, 041F0AG, 041F0AH, 041F0AJ, 041F0AK, 041F0AP, 041F0AQ,

	041F0J9, 041F0JB, 041F0JC, 041F0JD, 041F0JF, 041F0JG, 041F0JH, 041F0JJ, 041F0JK, 041F0JP, 041F0JQ, 041F0K9, 041F0KB, 041F0KC, 041F0KD, 041F0KF, 041F0KG, 041F0KH, 041F0KJ, 041F0KK, 041F0KP, 041F0KQ, 041F0Z9, 041F0ZB, 041F0ZC, 041F0ZD, 041F0ZF, 041F0ZG, 041F0ZH, 041F0ZJ, 041F0ZK, 041F0ZP, 041F0ZQ, 041F499, 041F49B, 041F49C, 041F49D, 041F49F, 041F49G, 041F49H, 041F49J, 041F49K, 041F49P, 041F49Q, 041F4A9, 041F4AB, 041F4AC, 041F4AD, 041F4AF, 041F4AG, 041F4AH, 041F4AJ, 041F4AK, 041F4AP, 041F4AQ, 041F4J9, 041F4JB, 041F4JC, 041F4JD, 041F4JF, 041F4JG, 041F4JH, 041F4JJ, 041F4JK, 041F4JP, 041F4JQ, 041F4K9, 041F4KB, 041F4KC, 041F4KD, 041F4KF, 041F4KG, 041F4KH, 041F4KJ, 041F4KK, 041F4KP, 041F4KQ, 041F4Z9, 041F4ZB, 041F4ZC, 041F4ZD, 041F4ZF, 041F4ZG, 041F4ZH, 041F4ZJ, 041F4ZK, 041F4ZP, 041F4ZQ, 041H099, 041H09B, 041H09C, 041H09D, 041H09F, 041H09G, 041H09H, 041H09J, 041H09K, 041H09P, 041H09Q, 041H0A9, 041H0AB, 041H0AC, 041H0AD, 041H0AF, 041H0AG, 041H0AH, 041H0AJ, 041H0AK, 041H0AP, 041H0AQ, 041H0J9, 041H0JB, 041H0JC, 041H0JD, 041H0JF, 041H0JG, 041H0JH, 041H0JJ, 041H0JK, 041H0JP, 041H0JQ, 041H0K9, 041H0KB, 041H0KC, 041H0KD, 041H0KF, 041H0KG, 041H0KH, 041H0KJ, 041H0KK, 041H0KP, 041H0KQ, 041H0Z9, 041H0ZB, 041H0ZC, 041H0ZD, 041H0ZF, 041H0ZG, 041H0ZH, 041H0ZJ, 041H0ZK, 041H0ZP, 041H0ZQ, 041H499, 041H49B, 041H49C, 041H49D, 041J0JP, 041J0JQ, 041J0K9, 041J0KB, 041J0KC, 041J0KD, 041J0KF, 041J0KG, 041J0KH, 041J0KJ, 041J0KK, 041J0KP, 041J0KQ, 041J0Z9, 041J0ZB, 041J0ZC, 041J0ZD, 041J0ZF, 041J0ZG, 041J0ZH, 041J0ZJ, 041J0ZK, 041J0ZP, 041J0ZQ, 041J499, 041J49B, 041J49C, 041J49D, 041J49F, 041J49G, 041J49H, 041J49J, 041J49K, 041J49P, 041J49Q, 041J4A9, 041J4AB, 041J4AC, 041J4AD, 041J4AF, 041J4AG, 041J4AH, 041J4AJ, 041J4AK, 041J4AP, 041J4AQ, 041J4J9, 041J4JB, 041J4JC, 041J4JD, 041J4JF, 041J4JG, 041J4JH, 041J4JJ, 041J4JK, 041J4JP, 041J4JQ, 041J4K9, 041J4KB, 041J4KC, 041J4KD, 041J4KF, 041J4KG, 041J4KH, 041J4KJ, 041J4KK, 041J4KP, 041J4KQ, 041J4Z9, 041J4ZB, 041J4ZC, 041J4ZD, 041J4ZF, 041J4ZG, 041J4ZH, 041J4ZJ, 041J4ZK, 041J4ZP, 041J4ZQ, 041K09H, 041K09J, 041K09K, 041K09L, 041K09M, 041K09N, 041K09P, 041K09Q, 041K09S, 041K0AH, 041K0AJ, 041K0AK, 041K0AL, 041K0AM, 041K0AN, 041K0AP, 041K0AQ, 041K0AS, 041K0JH, 041K0JJ, 041K0JK, 041K0JL, 041K0JM, 041K0JN, 041K0JP, 041K0JQ, 041K0JS, 041K0KH, 041K0KJ, 041K0KK, 041K0KL, 041K0KM, 041K0KN,

© 2025 Dhar G et al. *JAMA Network Open*

	041K0KP, 041K0KQ, 041K0KS, 041K0ZH, 041K0ZJ, 041K0ZK, 041K0ZL, 041K0ZM, 041K0ZN, 041K0ZP, 041K0ZQ, 041K0ZS, 041K49H, 041K49J, 041K49K, 041K49L, 041K49M, 041K49N, 041K49P, 041K49Q, 041K49S, 041K4AH, 041K4AJ, 041K4AK, 041K4AL, 041K4AM, 041K4AN, 041K4AP, 041K4AQ, 041K4AS, 041K4JH, 041K4JJ, 041K4JK, 041K4JL, 041K4JM, 041K4JN, 041K4JP, 041K4JQ, 041K4JS, 041K4KH, 041K4KJ, 041K4KK,041K4KL, 041K4KM, 041K4KN, 041K4KP, 041K4KQ, 041K4KS, 041K4ZH, 041K4ZJ, 041K4ZK, 041K4ZL, 041K4ZM, 041K4ZN, 041K4ZP, 041K4ZQ, 041K4ZS, 041L09H, 041L09J, 041L09K, 041L09L, 041L09M, 041L09N, 041L09P, 041L09Q, 041L09S, 041L0AH, 041L0AJ, 041L0AK, 041L0AL, 041L0AM, 041L0AN, 041L0AP, 041L0AQ, 041L0AS, 041L0JH, 041L0JJ, 041L0JK, 041L0JL, 041L0JM, 041L0JN, 041L0JP, 041L0JQ, 041L0JS, 041L0KH, 041L0KJ, 041L0KK, 041L0KL, 041L0KM, 041L0KN, 041L0KP, 041L0KQ, 041L0KS, 041L0ZH, 041L0ZJ, 041L0ZK, 041L0ZL, 041L0ZM, 041L0ZN, 041L0ZP, 041L0ZQ, 041L0ZS, 041L49H, 041L49J, 041L49K, 041L49L, 041L49M, 041L49N, 041L49P, 041L49Q, 041L49S, 041L4AH, 041L4AJ, 041L4AK, 041L4AL, 041L4AM, 041L4AN, 041L4AP, 041L4AQ, 041L4AS, 041L4JH, 041L4JJ, 041L4JK, 041L4JL, 041L4JM, 041L4JN, 041L4JP, 041L4JQ, 041L4JS, 041L4KH, 041L4KJ, 041L4KK, 041L4KL, 041L4KM, 041L4KN, 041L4KP, 041L4KQ, 041L4KS, 041L4ZH, 041L4ZJ, 041L4ZK, 041L4ZL, 041L4ZM, 041L4ZN, 041L4ZP, 041L4ZQ, 041L4ZS, 041M09L, 041M09M, 041M09P, 041M09Q, 041M09S, 041M0AL, 041M0AM, 041M0AP, 041M0AQ, 041M0AS, 041M0JL, 041M0JM, 041M0JP, 041M0JQ, 041M0JS, 041M0KL, 041M0KM, 041M0KP, 041M0KQ, 041M0KS, 041M0ZL, 041M0ZM, 041M0ZP, 041M0ZQ, 041M0ZS, 041M49L, 041M49M, 041M49P, 041M49Q, 041M49S, 041M4AL, 041M4AM, 041M4AP, 041M4AQ, 041M4AS, 041M4JL, 041M4JM, 041M4JP, 041M4JQ, 041M4JS, 041M4KL, 041M4KM, 041M4KP, 041M4KQ, 041M4KS, 041M4ZL, 041M4ZM, 041M4ZP, 041M4ZQ, 041M4ZS, 041N09L, 041N09M, 041N09P, 041N09Q, 041N09S, 041N0AL, 041N0AM, 041N0AP, 041N0AQ, 041N0AS, 041N0JL, 041N0JM, 041N0JP, 041N0JQ, 041N0JS, 041N0KL, 041N0KM, 041N0KP, 041N0KQ, 041N0KS, 041N0ZL, 041N0ZM, 041N0ZP, 041N0ZQ, 041N0ZS, 041N49L, 041N49M, 041N49P, 041N49Q, 041N49S, 041N4AL, 041N4AM, 041N4AP, 041N4AQ, 041N4AS, 041N4JL, 041N4JM, 041N4JP, 041N4JQ, 041N4JS, 041N4KL, 041N4KM, 041N4KP, 041N4KQ, 041N4KS, 041N4ZL, 041N4ZM, 041N4ZP, 041N4ZQ, 041N4ZS, 041T09P, 041T09Q, 041T09S, 041T0AP, 041T0AQ,

© 2025 Dhar G et al. *JAMA Network Open*

	041T0AS, 041T0JP, 041T0JQ, 041T0JS, 041T0KP, 041T0KQ, 041T0KS, 041T0ZP, 041T0ZQ, 041T0ZS, 041T49P, 041T49Q, 041T49S, 041T4AP, 041T4AQ, 041T4AS, 041T4JP, 041T4JQ, 041T4JS, 041T4KP, 041T4KQ, 041T4KS, 041T4ZP, 041T4ZQ, 041T4ZS, 041U09P, 041U09Q, 041U09S, 041U0AP, 041U0AQ, 041U0AS, 041U0JP, 041U0JQ, 041U0JS, 041U0KP, 041U0KQ, 041U0KS, 041U0ZP, 041U0ZQ, 041U0ZS, 041U49P, 041U49Q, 041U49S, 041U4AP, 041U4AQ, 041U4AS, 041U4JP, 041U4JQ, 041U4JS, 041U4KP, 041U4KQ, 041U4KS, 041U4ZP, 041U4ZQ, 041U4ZS, 041V09P, 041V09Q, 041V09S, 041V0AP, 041V0AQ, 041V0AS, 041V0JP, 041V0JQ, 041V0JS, 041V0KP, 041V0KQ, 041V0KS, 041V0ZP, 041V0ZQ, 041V0ZS, 041V49P, 041V49Q, 041V49S, 041V4AP, 041V4AQ, 041V4AS, 041V4JP, 041V4JQ, 041V4JS, 041V4KP, 041V4KQ, 041V4KS, 041V4ZP, 041V4ZQ, 041V4ZS, 041W09P, 041W09Q, 041W09S, 041W0AP, 041W0AQ, 041W0AS, 041W0JP, 041W0JQ, 041W0JS, 041W0KP, 041W0KQ, 041W0KS, 041W0ZP, 041W0ZQ, 041W0ZS, 041W49P, 041W49Q, 041W49S, 041W4AP, 041W4AQ, 041W4AS, 041W4JP, 041W4JQ, 041W4JS, 041W4KP, 041W4KQ, 041W4KS, 041W4ZP, 041W4ZQ, 041W4ZS, 047004Z, 047005Z, 047006Z, 047007Z, 04700D1, 04700D6, 04700DZ, 04700EZ, 04700F6, 04700FZ, 04700G6, 04700GZ, 04700Z1, 04700Z6, 04700ZZ, 047037Z, 04703D1, 04703D6, 04703DZ, 04703EZ, 04703F6, 04703FZ, 04703G6, 04703GZ, 04703Z1, 04703Z6, 04703ZZ, 047044Z, 047045Z, 047046Z, 047047Z, 04704D1, 04704D6, 04704DZ, 04704EZ, 04704F6, 04704FZ, 04704G6, 04704GZ, 04704Z1, 04704Z6, 04704ZZ, 047104Z, 047105Z, 047106Z, 047107Z, 04710D1, 04710D6, 04710DZ, 04710EZ, 04710F6, 04710FZ, 04710G6, 04710GZ, 04710Z1, 04710Z6, 04710ZZ, 047134Z, 047135Z, 047136Z, 047137Z, 04713D1, 04713D6, 04713DZ, 04713EZ, 04713F6, 04713FZ, 04713G6, 04713GZ, 04713Z1, 04713Z6, 04713ZZ, 047144Z, 047145Z, 047146Z, 047147Z, 04714D1, 04714D6, 04714DZ, 04714EZ, 04714F6, 04714FZ, 04714G6, 04714GZ, 04714Z1, 04714Z6, 04714ZZ, 047204Z, 047205Z, 047206Z, 047207Z, 04720D1, 04720D6, 04720DZ, 04720EZ, 04720F6, 04720FZ, 04720G6, 04720GZ, 04720Z1, 04720Z6, 04720ZZ, 047234Z, 047235Z, 047236Z, 047237Z, 04723D1, 04723D6, 04723DZ, 04723EZ, 04723F6, 04723FZ, 04723G6, 04723GZ, 04723Z1, 04723Z6, 04723ZZ, 047244Z, 047245Z, 047246Z, 047247Z, 04724D1, 04724D6, 04724DZ, 04724EZ, 04724F6, 04724FZ, 04724G6, 04724GZ, 04724Z1, 04724Z6, 04724ZZ, 047304Z,047305Z,047306Z, 047307Z, 04730D1, 04730D6, 04730DZ, 04730EZ, 04730F6,

	04730FZ, 04730G6, 04730GZ, 04730Z1, 04730Z6, 04730ZZ,047334Z,047335Z,047336Z,047337Z, 04733D1, 04733D6, 04733DZ, 04733EZ, 04733F6, 04733FZ, 04733G6, 04733GZ, 04733Z1, 04733Z6, 04733ZZ, 047344Z, 047345Z, 047346Z, 047347Z, 04734D1, 04734D6, 04734DZ, 04734EZ, 04734F6, 04734FZ, 04734G6, 04734GZ, 04734Z1, 04734Z6, 04734ZZ, 047404Z,047405Z, 047406Z,047407Z, 04740D1, 04740D6, 04740DZ, 04740EZ, 04740F6, 04740FZ, 04740G6, 04740GZ, 04740Z1, 04740Z6, 04740ZZ, 047434Z, 047435Z, 047436Z, 047437Z, 04743D1, 04743D6, 04743DZ, 04743EZ, 04743F6, 04743FZ, 04743G6, 04743GZ, 04743Z1, 04743Z6, 04743ZZ, 047444Z, 047445Z, 047446Z, 047447Z, 04744D1, 04744D6, 04744DZ, 04744EZ, 04744F6, 04744FZ, 04744G6, 04744GZ, 04744Z1, 04744Z6, 04744ZZ, 047504Z, 047505Z, 047506Z, 047507Z, 04750D1, 04750D6, 04750DZ, 04750EZ, 04750F6, 04750FZ, 04750G6, 04750GZ, 04750Z1, 04750Z6, 04750ZZ, 047534Z, 047535Z, 047536Z, 047537Z, 04753D1, 04753D6, 04753DZ, 04753EZ, 04753F6, 04753FZ, 04753G6, 04753GZ, 04753Z1, 04753Z6, 04753ZZ, 047544Z, 047545Z, 047546Z, 047547Z, 04754D1, 04754D6, 04754DZ, 04754EZ, 04754F6, 04754FZ, 04754G6, 04754GZ, 04754Z1, 04754Z6, 04754ZZ,047604Z, 047605Z, 047606Z, 047607Z, 04760D1, 04760D6, 04760DZ, 04760EZ, 04760F6, 04760FZ, 04760G6, 04760GZ, 04760Z1, 04760Z6, 04760ZZ, 047634Z, 047635Z, 047636Z, 047637Z, 04763D1, 04763D6, 04763DZ, 04763EZ, 04763F6, 04763FZ, 04763G6, 04763GZ, 04763Z1, 04763Z6, 04763ZZ,047644Z,047645Z,047646Z, 047647Z, 04764D1, 04764D6, 04764DZ, 04764EZ, 04764F6, 04764FZ, 04764G6, 04764GZ, 04764Z1, 04764Z6, 04764ZZ, 047704Z,047705Z,047706Z, 047707Z, 04770D1, 04770D6, 04770DZ,04770EZ, 04770F6, 04770FZ, 04770G6, 04770GZ, 04770Z1, 04770Z6, 04770ZZ,047734Z,047735Z,047736Z, 047737Z, 04773D1, 04773D6, 04773DZ, 04773EZ, 04773F6, 04773FZ, 04773G6, 04773GZ, 04773Z1, 04773Z6, 04773ZZ, 047744Z,047745Z,047746Z,047747Z, 04774D1, 04774D6, 04774DZ,04774EZ, 04774F6, 04774FZ, 04774G6, 04774GZ, 04774Z1, 04774Z6, 04774ZZ,047804Z,047805Z,047806Z, 047807Z, 04780D1, 04780D6, 04780DZ, 04780EZ, 04780F6, 04780FZ, 04780G6, 04780GZ, 04780Z1, 04780Z6, 04780ZZ,047834Z, 047835Z,047835Z, 047837Z, 04783D1, 04783D6, 04783DZ,04783EZ, 04783F6, 04783FZ, 04783G6, 04783GZ, 04783Z1, 04783Z6, 04783ZZ,047844Z,047845Z,047846Z, 047847Z, 04784D1,

© 2025 Dhar G et al. *JAMA Network Open*

	04784D6, 04784DZ,04784EZ, 04784F6, 04784FZ, 04784G6, 04784GZ, 04784Z1, 04784Z6, 04784ZZ,047904Z,047905Z,047906Z, 047907Z, 04790D1, 04790D6, 04790DZ,04790EZ, 04790F6, 04790FZ, 04790G6, 04790GZ, 04790Z1, 04790Z6, 04790ZZ,047934Z,047935Z,047936Z,047937Z, 04793D1, 04793D6, 04793DZ, 04793EZ, 04793F6, 04793FZ, 04793G6, 04793GZ, 04793Z1, 04793Z6, 04793ZZ, 047944Z,047945Z, 047946Z,047947Z, 04794D1, 04794D6, 04794DZ, 04794F6, 04794FZ, 04794G6, 04794GZ, 04794Z1, 04794Z6, 04794ZZ, 047A041, 047A046, 047A04Z, 047A056, 047A05Z, 047A066, 047A06Z, 047A076, 047A07Z, 047A0D1, 047A0D6, 047A0DZ, 047A0E6, 047A0EZ, 047A0F6, 047A0FZ, 047A0G6, 047A0GZ, 047A0Z1, 047A0Z6, 047A0ZZ, 047A341, 047A346, 047A34Z, 047A356, 047A35Z, 047A366, 047A36Z, 047A376, 047A37Z, 047A3D1, 047A3D6, 047A3DZ, 047A3E6, 047A3EZ, 047A3F6, 047A3FZ, 047A3G6, 047A3GZ, 047A3Z1, 047A3Z6, 047A3ZZ, 047A441, 047A446, 047A44Z, 047A456, 047A45Z, 047A466, 047A46Z, 047A476, 047A47Z, 047A4D1, 047A4D6, 047A4DZ, 047A4E6,047A4EZ, 047A4F6, 047A4FZ, 047A4G6, 047A4GZ, 047A4Z1, 047A4Z6, 047A4ZZ, 047B041, 047B046, 047B04Z, 047B056, 047B05Z, 047B066, 047B06Z, 047B076, 047B07Z, 047B0D1, 047B0D6, 047B0DZ, 047B0E6, 047B0EZ, 047B0F6, 047B0FZ, 047B0G6, 047B0GZ, 047B0Z1, 047B0Z6, 047B0ZZ, 047B341, 047B346, 047B34Z, 047B356, 047B35Z, 047B366, 047B36Z, 047B376, 047B37Z, 047B3D1, 047B3D6, 047B3DZ, 047B3E6, 047B3EZ, 047B3F6, 047B3FZ, 047B3G6, 047B3GZ, 047B3Z1, 047B3Z6, 047B3ZZ, 047B441, 047B446, 047B44Z, 047B456, 047B45Z, 047B466, 047B46Z, 047B476, 047B47Z, 047B4D1, 047B4D6, 047B4DZ, 047B4E6, 047B4EZ, 047B4F6, 047B4FZ, 047B4G6, 047B4GZ, 047B4Z1, 047B4Z6, 047B4ZZ, 047C041, 047C046, 047C04Z, 047C056, 047C05Z, 047C066, 047C06Z, 047C076, 047C07Z, 047C0D1, 047C0D6, 047C0DZ, 047C0E6, 047C0EZ, 047C0F6, 047C0FZ, 047C0G6, 047C0GZ, 047C0Z1, 047C0Z6, 047C0ZZ, 047C341, 047C346, 047C34Z, 047C356, 047C35Z, 047C366, 047C36Z, 047C376, 047C37Z, 047C3D1, 047C3D6, 047C3DZ, 047C3E6, 047C3EZ, 047C3F6, 047C3FZ, 047C3G6, 047C3GZ, 047C3Z1, 047C3Z6, 047C3ZZ, 047C441, 047C446, 047C44Z, 047C456, 047C45Z, 047C466, 047C46Z, 047C476, 047C47Z, 047C4D1, 047C4D6, 047C4DZ, 047C4E6,047C4EZ, 047C4F6, 047C4FZ, 047C4G6, 047C4GZ, 047C4Z1, 047C4Z6, 047C4ZZ, 047D041, 047D046, 047D04Z, 047D056, 047D05Z, 047D066, 047D06Z, 047D076, 047D07Z,

© 2025 Dhar G et al. *JAMA Network Open*

	047D0D1, 047D0D6, 047D0DZ, 047D0E6, 047D0EZ, 047D0F6, 047D0FZ, 047D0G6, 047D0GZ, 047D0Z1, 047D0Z6, 047D0ZZ, 047D341, 047D346, 047D34Z, 047D356, 047D35Z, 047D366, 047D36Z, 047D376, 047D37Z, 047D3D1, 047D3D6, 047D3DZ, 047D3E6, 047D3EZ, 047D3F6, 047D3FZ, 047D3G6, 047D3GZ, 047D3Z1, 047D3Z6, 047D3ZZ, 047D441, 047D446, 047D44Z, 047D456, 047D45Z, 047D466, 047D46Z, 047D476, 047D47Z, 047D4D1, 047D4D6, 047D4DZ, 047D4E6, 047D4EZ, 047D4F6, 047D4FZ, 047D4G6, 047D4GZ, 047D4Z1, 047D4Z6, 047D4ZZ,047E04Z,047E05Z,047E06Z,047E07Z, 047E0D1, 047E0D6, 047E0DZ, 047E0E6, 047E0EZ, 047E0F6, 047E0FZ, 047E0G6, 047E0GZ, 047E0Z1, 047E0Z6, 047E0ZZ, 047E341, 047E346, 047E34Z, 047E356, 047E35Z, 047E366, 047E36Z, 047E376, 047E37Z, 047E3D1, 047E3D6, 047E3DZ, 047E3E6, 047E3EZ, 047E3F6, 047E3FZ, 047E3G6, 047E3GZ, 047E3Z1, 047E3Z6, 047E3ZZ, 047E441, 047E446, 047E44Z, 047E456, 047E45Z, 047E466, 047E46Z, 047E476, 047E47Z, 047E4D1, 047E4D6, 047E4DZ, 047E4E6, 047E4EZ, 047E4F6, 047E4FZ, 047E4G6, 047E4GZ, 047E4Z1, 047E4Z6, 047E4ZZ, 047F041, 047F046, 047F04Z, 047F056, 047F05Z, 047F066, 047F06Z, 047F076, 047F07Z, 047F0D1, 047F0D6, 047F0DZ, 047F0E6, 047F0EZ, 047F0F6, 047F0FZ, 047F0G6, 047F0GZ, 047F0Z1, 047F0Z6, 047F0ZZ, 047F341, 047F346, 047F34Z, 047F356, 047F35Z, 047F366, 047F36Z, 047F376, 047F37Z, 047F3D1, 047F3D6, 047F3DZ, 047F3E6, 047F3EZ, 047F3F6, 047F3FZ, 047F3G6, 047F3GZ, 047F3Z1, 047F3Z6, 047F3ZZ, 047F441, 047F446, 047F44Z, 047F456, 047F45Z, 047F466, 047F46Z, 047F476, 047F47Z, 047F4D1, 047F4D6, 047F4DZ, 047F4E6,047F4EZ, 047F4F6, 047F4FZ, 047F4G6, 047F4GZ, 047F4Z1, 047F4Z6, 047F4ZZ, 047H041, 047H046, 047H04Z, 047H056, 047H05Z, 047H066, 047H06Z, 047H076, 047H07Z, 047H0D1, 047H0D6, 047H0DZ, 047H0E6, 047H0EZ, 047H0F6, 047H0FZ, 047H0G6, 047H0GZ, 047H0Z1, 047H0Z6, 047H0ZZ, 047H341, 047H346, 047H34Z, 047H356, 047H35Z, 047H366, 047H36Z, 047H376, 047H37Z, 047H3D1, 047H3D6, 047H3DZ, 047H3E6, 047H3EZ, 047H3F6, 047H3FZ, 047H3G6, 047H3GZ, 047H3Z1, 047H3Z6, 047H3ZZ, 047H441, 047H446, 047H44Z, 047H456, 047H45Z, 047H466, 047H46Z, 047H476, 047H47Z, 047H4D1, 047H4D6, 047H4DZ, 047H4E6, 047H4EZ, 047H4F6, 047H4FZ, 047H4G6, 047H4GZ, 047H4Z1, 047H4Z6, 047H4ZZ, 047J041, 047J046, 047J04Z, 047J056, 047J05Z, 047J066, 047J06Z, 047J076, 047J07Z, 047J0D1, 047J0D6, 047J0DZ, 047J0E6, 047J0EZ, 047J0F6, 047J0FZ, 047J0G6, 047J0GZ, 047J0Z1,

© 2025 Dhar G et al. *JAMA Network Open*

	047J0Z6, 047J0ZZ, 047J341, 047J346, 047J34Z, 047J356, 047J35Z, 047J366, 047J36Z, 047J376, 047J37Z, 047J3D1, 047J3D6, 047J3DZ, 047J3E6, 047J3EZ, 047J3F6, 047J3FZ, 047J3G6, 047J3GZ, 047J3Z1, 047J3Z6, 047J3ZZ, 047J441, 047J446, 047J44Z, 047J456, 047J45Z, 047J466, 047J46Z, 047J476, 047J47Z, 047J4D1, 047J4D6, 047J4DZ, 047J4E6, 047J4EZ, 047J4F6, 047J4FZ, 047J4G6, 047J4GZ, 047J4Z1, 047J4Z6, 047J4ZZ, 047K041, 047K046, 047K04Z, 047K056, 047K05Z, 047K066, 047K06Z, 047K076, 047K07Z, 047K0D1, 047K0D6, 047K0DZ, 047K0E6, 047K0EZ, 047K0F6, 047K0FZ, 047K0G6, 047K0GZ, 047K0Z1, 047K0Z6, 047K0ZZ, 047K341, 047K346, 047K34Z, 047K356, 047K35Z, 047K366, 047K36Z, 047K376, 047K37Z, 047K3D1, 047K3D6, 047K3DZ, 047K3E6, 047K3EZ, 047K3F6, 047K3FZ, 047K3G6, 047K3GZ, 047K3Z1, 047K3Z6, 047K3ZZ, 047K441, 047K446, 047K44Z, 047K456, 047K45Z, 047K466, 047K46Z, 047K476, 047K47Z, 047K4D1, 047K4D6, 047K4DZ, 047K4E6, 047K4EZ, 047K4F6, 047K4FZ, 047K4G6, 047K4GZ, 047K4Z1, 047K4Z6, 047K4ZZ, 047L041, 047L046, 047L04Z, 047L056, 047L05Z, 047L066, 047L06Z, 047L076, 047L07Z, 047L0D1, 047L0D6, 047L0DZ, 047L0E6, 047L0EZ, 047L0F6, 047L0FZ, 047L0G6, 047L0GZ, 047L0Z1, 047L0Z6, 047L0ZZ, 047L341, 047L346, 047L34Z, 047L356, 047L35Z, 047L366, 047L36Z, 047L376, 047L37Z, 047L3D1, 047L3D6, 047L3DZ, 047L3E6, 047L3EZ, 047L3F6, 047L3FZ, 047L3G6, 047L3GZ, 047L3Z1, 047L3Z6, 047L3ZZ, 047L441, 047L446, 047L44Z, 047L456, 047L45Z, 047L466, 047L46Z, 047L476, 047L47Z, 047L4D1, 047L4D6, 047L4DZ, 047L4E6, 047L4EZ, 047L4F6, 047L4FZ, 047L4G6, 047L4GZ, 047L4Z1, 047L4Z6, 047L4ZZ, 047M041, 047M046, 047M04Z, 047M056, 047M05Z, 047M066, 047M06Z, 047M076, 047M07Z, 047M0D1, 047M0D6, 047M0DZ, 047M0E6, 047M0EZ, 047M0F6, 047M0FZ, 047M0G6, 047M0GZ, 047M0Z1, 047M0Z6, 047M0ZZ, 047M341, 047M346, 047M34Z, 047M356, 047M35Z, 047M366, 047M36Z, 047M376, 047M37Z, 047M3D1, 047M3D6, 047M3DZ, 047M3E6, 047M3EZ, 047M3F6, 047M3FZ, 047M3G6, 047M3GZ, 047M3Z1, 047M3Z6, 047M3ZZ, 047M441, 047M446, 047M44Z, 047M456, 047M45Z, 047M466, 047M46Z, 047M476, 047M47Z, 047M4D1, 047M4D6, 047M4DZ, 047M4E6, 047M4EZ, 047M4F6, 047M4FZ, 047M4G6, 047M4GZ, 047M4Z1, 047M4Z6, 047M4ZZ, 047N041, 047N046, 047N04Z, 047N056, 047N05Z, 047N066, 047N06Z, 047N076, 047N07Z, 047N0D1, 047N0D6, 047N0DZ, 047N0E6, 047N0EZ, 047N0F6, 047N0FZ, 047N0G6, 047N0GZ, 047N0Z1, 047N0Z6, 047N0ZZ, 047N341, 047N346, 047N34Z, 047N356, 047N35Z,

	047N366, 047N36Z, 047N376, 047N37Z, 047N3D1, 047N3D6, 047N3DZ, 047N3E6, 047N3EZ, 047N3F6, 047N3FZ, 047N3G6, 047N3GZ, 047N3Z1, 047N3Z6, 047N3ZZ, 047N441, 047N446, 047N44Z, 047N456, 047N45Z, 047N466, 047N46Z, 047N476, 047N47Z, 047N4D1, 047N4D6, 047N4DZ, 047N4E6, 047N4EZ, 047N4F6, 047N4FZ, 047N4G6, 047N4GZ, 047N4Z1, 047N4Z6, 047N4ZZ, 047P041, 047P046, 047P04Z, 047P056, 047P05Z, 047P066, 047P06Z, 047P076, 047P07Z, 047P0D1, 047P0D6, 047P0DZ, 047P0E6, 047P0EZ, 047P0F6, 047P0FZ, 047P0G6, 047P0GZ, 047P0Z1, 047P0Z6, 047P0ZZ, 047P341, 047P346, 047P34Z, 047P356, 047P35Z, 047P366, 047P36Z, 047P376, 047P37Z, 047P3D1, 047P3D6, 047P3DZ, 047P3E6, 047P3EZ, 047P3F6, 047P3FZ, 047P3G6, 047P3GZ, 047P3Z1, 047P3Z6, 047P3ZZ, 047P441, 047P446, 047P44Z, 047P456, 047P45Z, 047P466, 047P46Z, 047P476, 047P47Z, 047P4D1, 047P4D6, 047P4DZ, 047P4E6, 047P4EZ, 047P4F6, 047P4FZ, 047P4G6, 047P4GZ, 047P4Z1, 047P4Z6,047P4ZZ, 047Q041, 047Q046, 047Q04Z, 047Q056, 047Q05Z, 047Q066, 047Q06Z, 047Q076, 047Q07Z, 047Q0D1, 047Q0D6, 047Q0DZ, 047Q0E6, 047Q0EZ, 047Q0F6, 047Q0FZ, 047Q0G6, 047Q0GZ, 047Q0Z1, 047Q0Z6, 047Q0ZZ, 047Q341, 047Q346, 047Q34Z, 047Q356, 047Q35Z, 047Q366, 047Q36Z, 047Q376, 047Q37Z, 047Q3D1, 047Q3D6, 047Q3DZ, 047Q3E6, 047Q3EZ, 047Q3F6, 047Q3FZ, 047Q3G6, 047Q3GZ, 047Q3Z1, 047Q3Z6, 047Q3ZZ, 047Q441, 047Q446, 047Q44Z, 047Q456, 047Q45Z, 047Q466, 047Q46Z, 047Q476, 047Q47Z, 047Q4D1, 047Q4D6, 047Q4DZ, 047Q4E6, 047Q4EZ, 047Q4F6, 047Q4FZ, 047Q4G6, 047Q4GZ, 047Q4Z1, 047Q4Z6, 047Q4ZZ, 047R041, 047R046, 047R04Z, 047R056, 047R05Z, 047R066, 047R06Z, 047R076, 047R07Z, 047R0D1, 047R0D6, 047R0DZ, 047R0E6, 047R0EZ, 047R0F6, 047R0FZ, 047R0G6, 047R0GZ, 047R0Z1, 047R0Z6, 047R0ZZ, 047R341, 047R346, 047R34Z, 047R356, 047R35Z, 047R366, 047R36Z,047R376, 047R37Z, 047R3D1, 047R3D6, 047R3DZ, 047R3E6, 047R3EZ, 047R3F6, 047R3FZ, 047R3G6, 047R3GZ, 047R3Z1, 047R3Z6, 047R3ZZ, 047R441, 047R446, 047R44Z, 047R456, 047R45Z, 047R466, 047R46Z, 047R476, 047R47Z, 047R4D1, 047R4D6, 047R4DZ, 047R4E6, 047R4EZ, 047R4F6, 047R4FZ, 047R4G6, 047R4GZ, 047R4Z1, 047R4Z6, 047R4ZZ, 047S041, 047S046, 047S04Z, 047S056, 047S05Z, 047S066, 047S06Z, 047S076, 047S07Z, 047S0D1, 047S0D6, 047S0DZ, 047S0E6, 047S0EZ, 047S0F6, 047S0FZ, 047S0G6, 047S0GZ, 047S0Z1, 047S0Z6, 047S0ZZ, 047S341, 047S346, 047S34Z, 047S356, 047S35Z, 047S366, 047S36Z, 047S376, 047S37Z, 047S3D1, 047S3D6,

	047S3DZ, 047S3E6, 047S3EZ, 047S3F6, 047S3FZ, 047S3G6, 047S3GZ, 047S3Z1, 047S3Z6, 047S3ZZ, 047S441, 047S446, 047S44Z, 047S456, 047S45Z, 047S466, 047S46Z, 047S476, 047S47Z,047S4D1, 047S4D6, 047S4DZ, 047S4E6, 047S4EZ, 047S4F6, 047S4FZ, 047S4G6, 047S4GZ, 047S4Z1, 047S4Z6, 047S4ZZ, 047T041, 047T046, 047T04Z, 047T056, 047T05Z, 047T066, 047T06Z, 047T076, 047T07Z, 047T0D1, 047T0D6, 047T0DZ, 047T0E6, 047T0EZ, 047T0F6, 047T0FZ, 047T0G6, 047T0GZ, 047T0Z1, 047T0Z6, 047T0ZZ, 047T341, 047T346, 047T34Z, 047T356, 047T35Z, 047T366, 047T36Z, 047T376, 047T37Z, 047T3D1, 047T3D6, 047T3DZ, 047T3E6, 047T3EZ, 047T3F6, 047T3FZ, 047T3G6, 047T3GZ, 047T3Z1, 047T3Z6, 047T3ZZ, 047T441, 047T446, 047T44Z, 047T456, 047T45Z, 047T466, 047T46Z,047T476, 047T47Z, 047T4D1, 047T4D6, 047T4DZ, 047T4E6, 047T4EZ, 047T4F6, 047T4FZ, 047T4G6, 047T4GZ, 047T4Z1, 047T4Z6, 047T4ZZ, 047U041, 047U046, 047U04Z, 047U056, 047U05Z, 047U066, 047U06Z, 047U076, 047U07Z, 047U0D1, 047U0D6, 047U0DZ, 047U0E6, 047U0EZ, 047U0F6, 047U0FZ, 047U0G6, 047U0GZ, 047U0Z1, 047U0Z6, 047U0ZZ, 047U341, 047U346, 047U34Z, 047U356, 047U35Z, 047U366, 047U36Z, 047U376, 047U37Z, 047U3D1, 047U3D6, 047U3DZ, 047U3E6, 047U3EZ, 047U3F6, 047U3FZ, 047U3G6, 047U3GZ, 047U3Z1, 047U3Z6, 047U3ZZ, 047U441, 047U446, 047U44Z, 047U456, 047U45Z, 047U466, 047U46Z, 047U476, 047U47Z, 047U4D1, 047U4D6, 047U4DZ, 047U4E6, 047U4EZ, 047U4F6, 047U4FZ, 047U4G6, 047U4GZ, 047U4Z1, 047U4Z6, 047U4ZZ, 047V041, 047V046, 047V04Z, 047V056, 047V05Z, 047V066, 047V06Z, 047V076, 047V07Z, 047V0D1, 047V0D6, 047V0DZ, 047V0E6, 047V0EZ, 047V0F6, 047V0FZ, 047V0G6, 047V0GZ, 047V0Z1, 047V0Z6, 047V0ZZ, 047V341, 047V346, 047V34Z, 047V356, 047V35Z, 047V366, 047V36Z, 047V376, 047V37Z, 047V3D1, 047V3D6,047V3DZ, 047V3E6, 047V3EZ, 047V3F6, 047V3FZ, 047V3G6, 047V3GZ, 047V3Z1, 047V3Z6, 047V3ZZ, 047V441, 047V446, 047V44Z, 047V456, 047V45Z, 047V466, 047V46Z, 047V476, 047V47Z, 047V4D1, 047V4D6, 047V4DZ, 047V4E6, 047V4EZ, 047V4F6, 047V4FZ, 047V4G6, 047V4GZ, 047V4Z1, 047V4Z6, 047V4ZZ, 047W041, 047W046, 047W04Z, 047W056, 047W05Z, 047W066, 047W06Z, 047W076, 047W07Z, 047W0D1, 047W0D6, 047W0DZ, 047W0E6, 047W0EZ, 047W0F6, 047W0FZ, 047W0G6, 047W0GZ, 047W0Z1, 047W0Z6, 047W0ZZ, 047W341, 047W346, 047W34Z, 047W356, 047W35Z, 047W366, 047W36Z, 047W376, 047W37Z, 047W3D1, 047W3D6, 047W3DZ, 047W3E6, 047W3EZ,

	047W3F6, 047W3FZ, 047W3G6, 047W3GZ, 047W3Z1, 047W3Z6, 047W3ZZ, 047W441, 047W446, 047W44Z, 047W456, 047W45Z, 047W466, 047W46Z, 047W476, 047W47Z, 047W4D1, 047W4D6, 047W4DZ, 047W4E6, 047W4EZ, 047W4F6, 047W4FZ, 047W4G6, 047W4GZ, 047W4Z1, 047W4Z6, 047W4ZZ, 047Y041, 047Y046, 047Y04Z, 047Y056, 047Y05Z, 047Y066, 047Y06Z, 047Y076, 047Y07Z, 047Y0D1, 047Y0D6, 047Y0DZ, 047Y0E6, 047Y0EZ, 047Y0F6, 047Y0FZ, 047Y0G6, 047Y0GZ, 047Y0Z1, 047Y0Z6, 047Y0ZZ, 047Y341, 047Y346, 047Y34Z, 047Y356, 047Y35Z, 047Y366, 047Y36Z, 047Y376, 047Y37Z, 047Y3D1, 047Y3D6, 047Y3DZ, 047Y3E6, 047Y3EZ, 047Y3F6, 047Y3FZ, 047Y3G6, 047Y3GZ, 047Y3Z1, 047Y3Z6, 047Y3ZZ, 047Y441, 047Y446, 047Y44Z, 047Y456, 047Y45Z, 047Y466, 047Y46Z, 047Y476, 047Y47Z, 047Y4D1, 047Y4D6, 047Y4DZ, 047Y4E6, 047Y4EZ, 047Y4F6, 047Y4FZ, 047Y4G6, 047Y4GZ, 047Y4Z1, 047Y4Z6, 047Y4ZZ, 04C10Z6, 04C10ZZ, 04C13Z6, 04C13ZZ, 04C14Z6, 04C14ZZ, 04C20Z6, 04C20ZZ, 04C23Z6, 04C23ZZ, 04C24Z6, 04C24ZZ, 04C30Z6, 04C30ZZ, 04C33Z6, 04C33ZZ, 04C34Z6, 04C34ZZ, 04C40Z6, 04C40ZZ, 04C43Z6, 04C43ZZ, 04C44Z6, 04C44ZZ, 04C50Z6, 04C50ZZ, 04C53Z6, 04C53ZZ, 04C54Z6, 04C54ZZ, 04C60Z6, 04C60ZZ, 04C63Z6, 04C63ZZ, 04C64Z6, 04C64ZZ, 04C70Z6, 04C70ZZ, 04C73Z6, 04C73ZZ, 04C74Z6, 04C74ZZ, 04C80Z6, 04C80ZZ, 04C83Z6, 04C83ZZ, 04C84Z6, 04C84ZZ, 04C90Z6, 04C90ZZ, 04C93Z6, 04C93ZZ, 04C94Z6, 04C94ZZ, 04CA0Z6, 04CA0ZZ, 04CA3Z6, 04CA3ZZ, 04CA4Z6, 04CA4ZZ, 04CB0Z6, 04CB0ZZ, 04CB3Z6, 04CB3ZZ, 04CB4Z6, 04CB4ZZ, 04CC0Z6, 04CC0ZZ, 04CC3Z6, 04CC3ZZ, 04CC4Z6, 04CC4ZZ, 04CD0Z6, 04CD0ZZ, 04CD3Z6,04CD3ZZ, 04CD4Z6, 04CD4ZZ, 04CE0Z6, 04CE0ZZ, 04CE3Z6, 04CE3ZZ, 04CE4Z6, 04CE4ZZ, 04CF0Z6, 04CF0ZZ, 04CF3Z6, 04CF3ZZ, 04CF4Z6, 04CF4ZZ, 04CH0Z6, 04CH0ZZ, 04CH3Z6, 04CH3ZZ, 04CH4Z6, 04CH4ZZ, 04CJ0Z6, 04CJ0ZZ, 04CJ3Z6, 04CJ3ZZ, 04CJ4Z6, 04CJ4ZZ, 04CK0Z6, 04CK0ZZ, 04CK3Z6, 04CK3ZZ, 04CK4Z6, 04CK4ZZ, 04CL0Z6, 04CL0ZZ, 04CL3Z6, 04CL3ZZ, 04CL4Z6, 04CL4ZZ, 04CM0Z6, 04CM0ZZ, 04CM3Z6, 04CM3ZZ, 04CM4Z6, 04CM4ZZ, 04CN0Z6, 04CN0ZZ, 04CN3Z6, 04CN3ZZ, 04CN4Z6, 04CN4ZZ, 04CP0Z6, 04CP0ZZ, 04CP3Z6, 04CP3ZZ, 04CP4Z6, 04CP4ZZ, 04CQ0Z6, 04CQ0ZZ, 04CQ3Z6, 04CQ3ZZ, 04CQ4Z6, 04CQ4ZZ, 04CR0Z6, 04CR0ZZ, 04CR3Z6, 04CR3ZZ, 04CR4Z6, 04CR4ZZ, 04CS0Z6, 04CS0ZZ, 04CS3Z6, 04CS3ZZ, 04CS4Z6, 04CS4ZZ, 04CT0Z6, 04CT0ZZ, 04CT3Z6, 04CT3ZZ, 04CT4Z6, 04CT4ZZ, 04CU0Z6, 04CU0ZZ, 04CU3Z6, 04CU3ZZ, 04CU4Z6, 04CU4ZZ, 04CV0Z6, 04CV0ZZ,

	04CV3Z6, 04CV3ZZ, 04CV4Z6, 04CV4ZZ, 04CW0Z6, 04CW0ZZ, 04CW3Z6, 04CW3ZZ, 04CW4Z6, 04CW4ZZ, 04CY0Z6, 04CY0ZZ, 04CY3Z6, 04CY3ZZ, 04CY4Z6, 04CY4ZZ, 051007Y, 051009Y, 05100AY, 05100JY, 05100KY, 05100ZY, 051047Y, 051049Y, 05104AY, 05104JY, 05104KY, 05104ZY, 051107Y, 051109Y, 05110AY, 05110JY, 05110KY, 05110ZY, 051147Y, 051149Y, 05114AY, 05114JY, 05114KY, 05114ZY, 051307Y, 051309Y, 05130AY, 05130JY, 05130KY, 05130ZY, 051347Y, 051349Y, 05134AY, 05134JY, 05134KY, 05134ZY, 051407Y, 051409Y, 05140AY, 05140JY, 05140KY, 05140ZY, 051447Y, 051449Y, 05144AY, 05144JY, 05144KY, 05144ZY, 051507Y, 051509Y, 05150AY, 05150JY, 05150KY, 05150ZY, 051547Y, 051549Y, 05154AY, 05154JY, 05154KY, 05154ZY, 051607Y, 051609Y, 05160AY, 05160JY, 05160KY, 05160ZY, 051647Y, 051649Y, 05164AY, 05164JY, 05164KY, 05164ZY, 051707Y, 051709Y, 05170AY,051A0JY, 051A0KY, 051A0ZY, 051A47Y, 051A49Y, 051A4AY, 051A4JY, 051A4KY, 051A4ZY, 051B07Y, 051B09Y, 051B0AY, 051B0JY, 051B0KY, 051B0ZY, 051B47Y, 051B49Y, 051B4AY, 051B4JY, 051B4KY, 051B4ZY, 051C07Y, 051C09Y, 051C0AY, 051C0JY, 051C0KY, 051C0ZY, 051C47Y, 051C49Y, 051C4AY, 051C4JY, 051C4KY, 051C4ZY, 051D07Y, 051D09Y, 051D0AY, 051D0JY, 051D0KY, 051D0ZY, 051D47Y, 051D49Y, 051D4AY, 051D4JY, 051D4KY, 051D4ZY, 051F07Y, 051F09Y, 051F0AY, 051F0JY, 051F0KY, 051F0ZY, 051F47Y, 051F49Y, 051F4AY, 051F4JY, 051F4KY, 051F4ZY, 051G07Y, 051G09Y, 051G0AY, 051G0JY, 051G0KY, 051G0ZY, 051G47Y, 051G49Y, 051G4AY, 051G4JY, 051G4KY, 051G4ZY, 051H07Y, 051H09Y, 051H0AY, 051H0JY, 051H0KY, 051H0ZY, 051H47Y, 051H49Y, 051H4AY, 051H4JY, 051H4KY, 051H4ZY, 051L07Y, 051L09Y, 051L0AY, 051L0JY, 051L0KY, 051L0ZY, 051L47Y, 051L49Y, 051L4AY, 051L4JY, 051L4KY, 051L4ZY, 051M07Y, 051M09Y, 051M0AY, 051M0JY, 051M0KY, 051M0ZY, 051M47Y, 051M49Y, 051M4AY, 051M4JY, 051M4KY, 051M4ZY, 051N07Y, 051N09Y, 051N0AY, 051N0JY, 051N0KY, 051N0ZY, 051N47Y, 051N49Y, 051N4AY, 051N4JY, 051N4KY, 051N4ZY, 051P07Y, 051P09Y, 051P0AY, 051P0JY, 051P0KY, 051P0ZY, 051P47Y, 051P49Y, 051P4AY, 051P4JY, 051P4KY, 051P4ZY, 051Q07Y, 051Q09Y, 051Q0AY, 051Q0JY, 051Q0KY, 051Q0ZY, 051Q47Y, 051Q49Y, 051Q4AY, 051Q4JY, 051Q4KY, 051Q4ZY, 051T07Y, 051T09Y, 051T0AY, 051T0JY, 051T0KY, 051T0ZY, 051T47Y, 051T49Y, 051T4AY, 051T4JY, 051T4KY, 051T4ZY, 051V07Y, 051V09Y, 051V0AY, 051V0JY, 051V0KY, 051V0ZY, 051V47Y, 051V49Y, 051V4AY, 051V4JY,

	051V4KY, 051V4ZY, 05700DZ, 05700ZZ, 05703DZ, 05703ZZ, 05704DZ, 05704ZZ, 05710DZ, 05710ZZ, 05713DZ, 05713ZZ, 05714DZ, 05714ZZ, 05720DZ, 05720ZZ, 05723DZ, 05723ZZ, 05724DZ, 05724ZZ, 05730DZ, 05730ZZ, 05733DZ, 05733ZZ, 05734DZ, 05734ZZ, 05740DZ, 05740ZZ, 05743DZ, 05743ZZ, 057C3ZZ, 057C4DZ, 057C4ZZ, 057D0DZ, 057D0ZZ, 057D3DZ, 057D3ZZ, 057D4DZ, 057D4ZZ, 057F0DZ, 057F0ZZ, 057F3DZ, 057F3ZZ, 057F4DZ, 057F4ZZ, 057G0DZ, 057G0ZZ, 057G3DZ, 057G3ZZ, 057G4DZ, 057G4ZZ, 057H0DZ, 057H0ZZ, 057H3DZ, 057H3ZZ, 057H4DZ, 057H4ZZ, 057L0DZ, 057L0ZZ, 057L3ZZ, 057L4ZZ, 057M0DZ, 057M0ZZ, 057M3ZZ, 057M4ZZ, 057N0DZ, 057N0ZZ, 057N3ZZ, 057N4ZZ, 057P0DZ, 057P0ZZ, 057P3ZZ, 057P4ZZ, 057Q0DZ, 057Q0ZZ, 057Q3ZZ, 057Q4ZZ, 057R0DZ, 057R0ZZ, 057R3ZZ, 057R4ZZ, 057S0DZ, 057S0ZZ, 057S3ZZ, 057S4ZZ, 057T0DZ, 057T0ZZ, 057T3DZ, 057T3ZZ, 057T4DZ, 057T4ZZ, 057V0DZ, 057V0ZZ, 057V3DZ, 057V3ZZ, 057V4DZ, 057V4ZZ, 057Y0DZ, 057Y0ZZ, 057Y3DZ, 057Y3ZZ, 057Y4DZ, 057Y4ZZ, 061307Y, 061309Y, 06130AY, 06130JY, 06130KY, 06130ZY, 061347Y, 061349Y, 06134AY, 06134JY, 06134KY, 06134ZY, 061C07Y, 061C09Y, 061C0AY, 061C0JY, 061C0KY, 061C0ZY, 061C47Y, 061C49Y, 061C4AY, 061C4JY, 061C4KY, 061C4ZY, 061D07Y, 061D09Y, 061D0AY, 061D0JY, 061D0KY, 061D0ZY, 061D47Y, 061D49Y, 061D4AY, 061D4JY, 061D4KY, 061D4ZY, 061F07Y, 061F09Y, 061F0AY, 061F0JY, 061F0KY, 061F0ZY, 061F47Y, 061F49Y, 061F4AY, 061F4JY, 061F4KY, 061F4ZY, 061G07Y, 061G09Y, 061G0AY, 061G0JY, 061G0KY, 061G0ZY, 061G47Y, 061G49Y, 061G4AY, 061G4JY, 061G4KY, 061G4ZY, 061H07Y, 061H09Y, 061H0AY, 061H0JY, 061H0KY, 061H0ZY, 061H47Y, 061H49Y, 061H4AY, 061H4JY, 061H4KY, 061H4ZY, 061M07Y, 061M09Y, 061M0AY, 061M0JY, 061M0KY, 061M0ZY, 061M47Y, 061M49Y, 061M4AY, 061M4JY, 061M4KY, 061M4ZY, 061N07Y, 061N09Y, 061N0AY, 061N0JY, 061N0KY, 061N0ZY, 061N47Y, 061N49Y, 061N4AY, 061N4JY, 061N4KY, 061N4ZY, 061P07Y, 061P09Y, 061P0AY, 061P0JY, 061P0KY, 061P0ZY, 061P47Y, 061P49Y, 061P4AY, 061P4JY, 061P4KY, 061P4ZY, 061Q07Y, 061Q09Y, 061Q0AY, 061Q0JY, 061Q0KY, 061Q0ZY, 061Q47Y, 061Q49Y, 061Q4AY, 061Q4JY, 061Q4KY, 061Q4ZY, 061T07Y, 061T09Y, 061T0AY, 061T0JY, 061T0KY, 061T0ZY, 061T47Y, 061T49Y, 061T4AY, 061T4JY, 061T4KY, 061T4ZY, 061V07Y, 061V09Y, 061V0AY, 061V0JY, 061V0KY, 061V0ZY, 061V47Y, 061V49Y, 061V4AY, 061V4JY, 061V4KY, 061V4ZY, 06700DZ, 06700ZZ, 06703DZ, 06703ZZ, 06704DZ, 06704ZZ, 06710DZ, 06710ZZ, 06713DZ,

	06713ZZ, 06714DZ, 06714ZZ, 06720DZ, 06720ZZ, 06723DZ, 06723ZZ, 06724DZ, 06724ZZ, 06730DZ, 06730ZZ, 06733DZ, 06733ZZ, 06734DZ, 06734ZZ, 06740DZ, 06740ZZ, 06743DZ, 06743ZZ, 06744DZ, 06744ZZ, 06750DZ, 06750ZZ, 06753DZ, 06753ZZ, 06754DZ, 06754ZZ, 06760DZ, 06760ZZ, 06763DZ, 06763ZZ, 06764DZ, 06764ZZ, 06770DZ, 06770ZZ, 06773DZ, 06773ZZ, 06774DZ, 06774ZZ, 06780DZ, 06780ZZ, 06783DZ, 06783ZZ, 06784DZ, 06784ZZ, 06790DZ, 06790ZZ, 06793DZ, 06793ZZ, 06794DZ, 06794ZZ, 067A0DZ, 067A0ZZ, 067A3DZ, 067A3ZZ, 067A4DZ, 067A4ZZ, 067B0DZ, 067B0ZZ, 067B3DZ, 067B3ZZ, 067B4DZ, 067B4ZZ, 067C0DZ, 067C0ZZ, 067C3DZ,067C3ZZ, 067C4DZ, 067C4ZZ, 067D0DZ, 067D0ZZ, 067D3DZ, 067D3ZZ, 067D4DZ, 067D4ZZ, 067F0DZ, 067F0ZZ, 067F3DZ, 067F3ZZ, 067F4DZ, 067F4ZZ, 067G0DZ, 067G0ZZ, 067G3DZ, 067G3ZZ, 067G4DZ, 067G4ZZ, 067H0DZ, 067H0ZZ, 067H3DZ, 067H3ZZ, 067H4DZ, 067H4ZZ, 067J0DZ, 067J0ZZ, 067J3DZ, 067J3ZZ, 067J4DZ, 067J4ZZ, 067M0DZ, 067M0ZZ, 067M3DZ, 067M3ZZ, 067M4DZ, 067M4ZZ, 067N0DZ, 067N0ZZ, 067N3DZ, 067N3ZZ, 067N4DZ, 067N4ZZ, 067P0DZ, 067P0ZZ, 067P3DZ, 067P3ZZ, 067P4DZ, 067P4ZZ, 067Q0DZ, 067Q0ZZ, 067Q3DZ, 067Q3ZZ, 067Q4DZ, 067Q4ZZ, 067T0DZ, 067T0ZZ, 067T3DZ, 067T3ZZ, 067T4DZ, 067T4ZZ, 067V0DZ, 067V0ZZ, 067V3DZ, 067V3ZZ, 067V4DZ, 067V4ZZ, 067Y0DZ, 067Y0ZZ, 067Y3DZ, 067Y3ZZ, 067Y4DZ, 067Y4ZZ, 312090-312099, 313090-313099, 314090-314099, 315090-315099, 316090-316099, 317090, 317093, 318091, 318094, 319093, 370056, 370056, 370056, 370066, 370076, 370346, 370356, 370366, 370376, 370446, 370456, 370466, 370476, 371046, 371056, 371066, 371076, 371346, 371356, 371366, 371376, 371446, 371456, 371466, 371476, 372046, 372056, 372066, 372076, 372346, 372356, 372366, 372376, 372446, 372456, 372466, 372476, 373046, 373056, 373066, 373076, 373346, 373356, 373366, 373376, 373446, 373456, 373466, 373476, 374046, 374056, 374066, 374076, 374346, 374356, 374366, 374376, 374446, 374456, 374466, 374476, 375046, 375056, 375066, 375076, 375346, 375356, 375366, 375376, 375446, 375456, 375466, 375476, 376046, 376056, 376066, 376076, 376346, 376356, 376366, 376376, 376446, 376456, 376466, 376476, 377046, 377056, 377066, 377076, 377346, 377356, 377366, 377376, 377446, 377456,377466, 377476, 378046, 378056, 378066, 378076, 378346, 378356, 378366, 378376, 378446, 378456, 378466, 378476, 379046, 379056, 379066, 379076, 379346, 379356, 379366, 379376, 379446, 379456, 379466, 379476,

	410090-410099, 410490-410499, 413093-413095, 413493-413495, 414093-414095, 414493-414495, 470041, 470046, 470056, 470066, 470076, 470341, 470346, 470356, 470366, 470376, 470441, 470446, 470456, 470466, 470476, 471041, 471046, 471056, 471066, 471076, 471441, 471446, 471456, 471466, 471476, 472041, 472046, 472056, 472066, 472076, 472341, 472346, 472356, 472366, 472376, 472441, 472446, 472456, 472466, 472476, 473041, 473046, 473056, 473066, 473076, 473341, 473346, 473356, 473366, 473376, 473441, 473446, 473456, 473466 , 473476, 474041, 474046, 474056, 474066, 474076, 474341, 474346, 474356, 474366, 474376, 474441, 474446, 474456, 474466, 474476, 475041, 475046, 475056, 475066, 475076, 475341, 475346, 475356, 475366, 475376, 475441, 475446, 475456, 475466, 475476, 476041, 476046, 476056, 476066, 476076, 476341, 476346, 476356, 476366, 476376, 476441, 476446, 476456, 476466, 476476, 477041, 477046, 477056, 477066, 477076, 477341, 477346, 477356, 477366, 477376, 477441, 477446, 477456, 477466, 477476, 478041, 478046, 478056, 478066, 478076, 478341, 478346, 478356, 478366, 478376, 478441, 478446, 478456, 478466, 478476, 479041, 479046, 479056, 479066, 479076, 479341, 479346, 479356, 479366, 479376, 479441, 479446, 479456, 479466, 479476, 3700000000,3703000000, 3704000000, 3710000000, 3713000000, 3714000000,3720000000, 3723000000, 3724000000, 3730000000, 3733000000,3734000000, 3740000000, 3743000000, 3744000000, 3750000000,3753000000, 3754000000, 3760000000, 3763000000,3764000000, 3770000000, 3773000000, 3774000000, 3780000000, 3783000000,3784000000, 3790000000, 3793000000, 3794000000, 4700000000, 4703000000, 4704000000, 4710000000, 4714000000, 4720000000, 4723000000, 4724000000, 4730000000, 4733000000,4734000000, 4740000000, 4743000000, 4744000000, 4750000000, 4753000000, 4754000000, 4760000000, 4763000000, 4764000000, 4770000000, 4773000000, 4774000000, 4780000000, 4783000000, 4784000000, 4790000000, 4793000000, 4794000000
**Concomitant medications**	Non-steroidal anti-inflammatory drugs, Antiplatelet drugs, Angiotensinogen converting enzyme inhibitor, Aldosterone Receptor Antagonists, Angiotensin receptor blocker, Beta Blockers, Calcium Channel Blockers, Proton Pump Inhibitors, Statins

© 2025 Dhar G et al. *JAMA Network Open*

eTable 2. Codes Used to Define Outcomes

Outcome	Information used to ascertain outcome
Bleeding events	ICD-10-CM codes. D62, I312, I602, I604, I606-616, I618-619, I621, I629, I6000-6002, I6010-6012, I6030-6032, I6050-6052, I6200-6203, K250, K252, K254, K256, K260, K262, K264, K266, K270, K272, K274, K276, K280, K282, K284, K286, K625, K920-922, K5521, K5701, K5711, K5713, K5721, K5731, K5733, K5741, K5751, K5753, K5781, K5791, K5793, K56699, M2500, M2508, M25011, M25012, M25019, M25021, M25022, M25029, M25031, M25032, M25039, M25041, M25042, M25049, M25051, M25052, M25059, M25061, M25062, M25069, M25071-25076, R040, R042, R049, R0481, R0489, R58
Non-fatal stroke	ICD-10-CM codes. I2601, I2602, I2609, I2690, I2692, I2699, I7401, I7409, I7410, I7411, I7419,I742, I743, I744, I745, I748, I749, T800XXA, T81718A, T8172XA, T82817A, T82818A

We began our work with diagnosis and procedure codes specified in the Forward-backward mapping schema defined by Webster-Clark 2020. **Codes** for outcomes were further adjudicated by an expert in health information management and medical coding.

© 2025 Dhar G et al. *JAMA Network Open*

eTable 3. Rationale and Justification of Criteria Used for Propensity Matching.

There was a lot of discussion during analysis between clinician scientists (nephrologist Nishank Jain, cardiologist Gaurav Dhar), pharmacoepidemiologist (Theresa Shireman), and expert statisticians in USRDS data analyses (Milind Phadnis and Suzanne Hunt). Rationale for the 2 most important decisions in this analysis are now included **as eTable 3**. We also describe it here:

Rationale for choosing 1:7 matching. We hypothesized that there could be a survival benefit of LAAO use over OAC use due to reduced risk of bleeding from LAAO. This required us to model "recurrent bleeding events" and the terminal event (i.e., death) jointly. Additionally, there was also an "interim stroke effect" which needed to be accounted for; this was done by making interim stroke event as a time-dependent covariate. To include all these parameters and adjustment for other risk factors, we needed a large sample size. This meant to include as many LAAO subjects as possible. After applying inclusion and exclusion criteria, we had 293 LAAO recipients. We realize recent studies in the general population matched LAAO and OACs 1:1 using Medicare registry data. Reference # 10 (PMID 36780379) compared older patients with AF treated with OACs compared with LAAO. There were 4,085 LAAO recipients. This large sample size allowed them to match LAAO group 1:1 with OAC group. **However, most of the KF patients with AF do not receive risk reduction care for AF as much as the general population (reference #8).** We found only 293 LAAO subjects in the entire national dataset after inclusion and exclusion criteria were applied. Therefore, we had to explore various matching ratios with the goal to estimate all parameters in the joint frailty model. See examples of various matching ratios in the Table below:

Ratio of propensity matching	Number of LAAO vs number of OACs	Is adjustment for covariates possible?	Is adjustment for time-dependent "interim stroke" possible?	Is estimation of correlation between recurrent bleeds and death possible?	Is the Joint frailty model convergence stable?
1:2	293 vs 586	Yes	No	No	No
1:4	293 vs 1,172	Yes	Yes	No	No
1:6	293 vs 1,758	Yes	Yes	Yes	No
1:7	293 vs 2,051	Yes	Yes	Yes	Yes

There are previous publications using alternative matching ratios in other diseases (Reference # 15, PMID: 16632131). We were guided by this report from Sturmer *et al*. The authors performed a review of 190 observational studies in PubMed that used PS matching. The authors reported that "use of PS comes at the price of losing potentially useful information about outcome. It therefore seems desirable to use PS for a reduction in bias and/or an improvement in efficiency". Moreover, based on Austin paper as cited by this reviewer, a bias can be introduced only if good

matches are not found. In our case, we found good matches even with a 1:7 matching strategy; standardized differences in baseline CHA_2DS_2-VASc score was 0.

Rationale for choosing 2 risk factors for propensity matching. CHA_2DS_2-VASc scoring system includes scores for congestive heart failure, hypertension, age ≥75 years, age 65-74 years, diabetes, stroke, vascular disease, female sex. Instead of matching the two groups based on each risk factor as done in recent publication shown as reference #10 (PMID 36780379), we used CHA_2DS_2-VASc scoring system to propensity-match the two groups given sample size of LAAO recipients. Because clinical decisions are made for the choice of risk reduction care in patients with AF (clinical practice guidelines PMID: 38033089, 38043043) based on the scoring system, this decision is justified.

Moreover, we wanted to retain as many LAAO subjects as possible for the data at hand. If we chose to match each risk factor individually, we would have lost sample size to perform meaningful analysis. As described in the report from Sturmer *et al* (Reference #15), we were guided by the "to improve efficiency" principle to decide about "what" to use for PS matching. Other individual risk factors (e.g., race, age, others) were not included in propensity matching but adjusted in the model. We do not expect standardized differences to be <10% for these factors. We would like to re-emphasize that we followed the dataset at hand and chose a well-reasoned and rigorous approach according to the data. If the editors and the reviewers allow, we could remove standardized differences of unmatched risk factors from Table 1 to avoid misinterpretation.

© 2025 Dhar G et al. *JAMA Network Open*

eTable 4. Rationale and Justification of the Joint Frailty Model Over Other Models including Fine and Gray Model.

Here, we briefly discuss the thought process leading to the choice of a *Joint Frailty Model* for analyzing our data. In doing so, we focus on the key research questions:<br>1. Is there an overall survival benefit comparing LAAO vs OAC when considering Death as the primary outcome?<br>2. Does LAAO reduce risk of recurrent bleeding compared to OAC? If so, does this translate to the overall survival benefit mentioned in #1 above?<br>3. Does LAAO reduce risk of first non-fatal stroke compared to OAC? If so, does this translate to the overall survival benefit mentioned in #1 above?<br>4. Are recurrent bleeding events independently associated with an increased risk of Death?<br>5. Does non-fatal stroke independently increase the risk of death?	To answer these questions, we consider the following model building approach in increasing order of complexity. We chose the final model that is most consistent with our data structure and that which enables us to answer the research questions in a practically meaningful way.
Model 1: A Fine & Gray competing risks model with "non-fatal stroke" and "death" acting as competing risks.<br><br>This is the classical "competing risks" scenario with "death" and "stroke" acting as competing risks.	Data Structure #1:<br>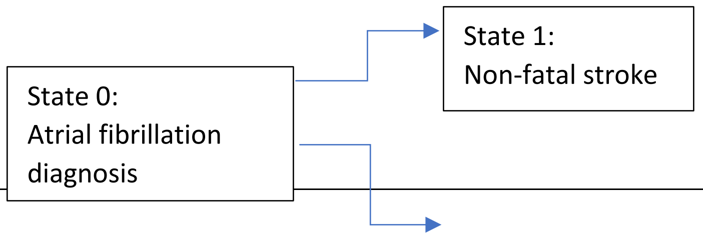

© 2025 Dhar G et al. *JAMA Network Open*

<table>
<tr>
<td>Note the lack of a directional arrow between State 1 “stroke” and State 2 “death” implying that only the first out of “time-to-stroke” and “time-to-death” can be observed. That is, when modeling “time-to-stroke”, we cannot treat “death” as merely another random censoring event and vice versa.

The Fine & Gray model is the right choice of statistical model to handle this scenario.</td>
<td>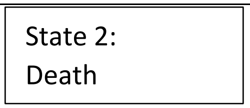

Here all subjects start with an initial state “Atrial fibrillation diagnosis” (State 0). Some of them experience a “non-fatal stroke” (State 1), some experience “death” (State 2), while others may be alive without a stroke at the end of the observation window.</td>
</tr>
<tr>
<td>Model 2: A multi-state model with “non-fatal stroke” and “death” that allows assessment of how occurrence of non-fatal stroke increases the risk of death.

Fact:

{i} A Cox PH model with a single time-dependent covariate is the same as analyzing a multi-state model with exactly 3 states.

{ii} The competing risks model is a special case of the more general topic of multi-state models.</td>
<td>Data Structure #2:

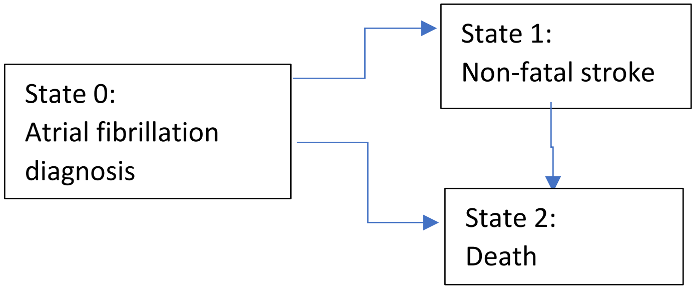

Here, the only difference compared to the first scenario is the directional arrow between “non-fatal stroke” and “death”. Thus, here we do possess data which will allow us a comparison of the type: ‘risk of death without stroke’ versus ‘risk of death with a non-fatal stroke’. Thus, when analyzing time-to-death, stroke (State 1) acts as an intermediary state between State 0 and State</td>
</tr>
</table>

© 2025 Dhar G et al. *JAMA Network Open*

<table>
<tr>
<td></td>
<td>2. This leads to the notion of “stroke as a time-dependent covariate” – some subjects start as “non-strokers”, but become “strokers” as some point in the observation window.</td>
</tr>
<tr>
<td>Model 3: A multi-state model with more intermediary states – that is a more complex system of more than one time-dependent covariate.

A multi-state model for time-to-death within the Cox PH framework can be used treating “stroke” and “number of bleeds” as time-dependent covariate. One limitation of doing so is that when modeling time-to-death, both stroke and bleeding events are being treated as covariates and not as (multivariate) outcomes.</td>
<td>Data Structure #3:
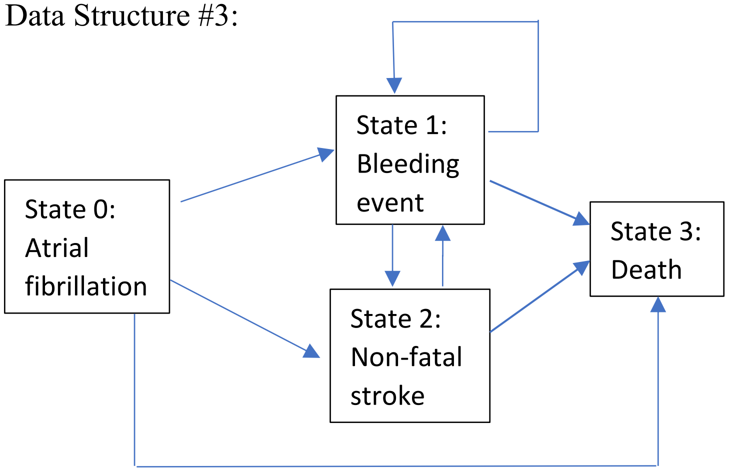

The figure above shows a more complex scenario with many possibilities. Subjects start at State 0 (Atrial fibrillation) and may or may not experience death in the observation window. They may or may not experience a non-fatal stroke prior to death. Likewise, they may or may not experience one or more bleeding events prior to death. The arrow that starts at State 1 and also ends at State 1 indicates the recurrent nature of bleeding events.</td>
</tr>
<tr>
<td colspan="2">Data Structure 4:</td>
</tr>
</table>

© 2025 Dhar G et al. *JAMA Network Open*

Adding another layer of complexity to Data Structure 3 to account for time-dependent treatment exposure leads to the following figure.

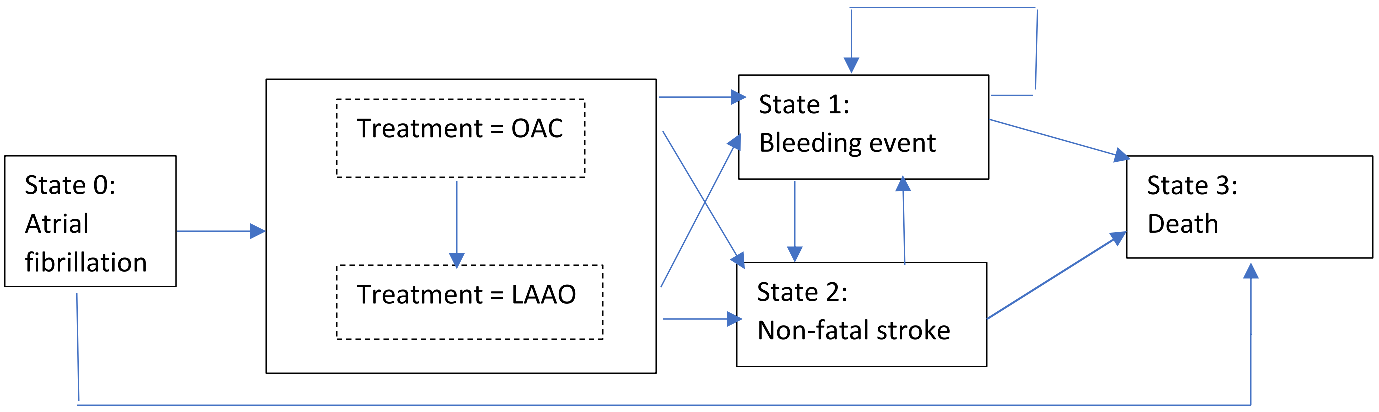

The figure above is the best representation of our data. That is treatment exposure (OAC or LAAO) is also a time-dependent covariate. That is, subjects diagnosed with atrial fibrillation start without any treatment. They are then assigned to OAC or LAAO. There is also a subset of subjects who get LAAO after spending some time taking OAC.

Stepwise approach to analyzing this data:<br><br>In view of the research questions and the structure of our data, we adopted the following stepwise approach to data analysis. At each step, modeling results were studied before proceeding to the next model.	Main Result
Step 1:<br><br>A Cox PH model for "Time-To-Death" is fit with –<br><br>{i} treatment exposure (LAAO vs OAC) as time-dependent covariate	LAAO reduces hazard of death compared to OAC (HR=0.71, p=0.0216) after adjusting for other risk factors. Non-fatal stroke independently increases the risk of death (HR=1.59, p<0.0001). Every new occurrence of a bleed increases the risk of death

© 2025 Dhar G et al. *JAMA Network Open*

{ii} non-fatal stroke as a time-dependent covariate,<br>{iii} number of bleeds as a time-dependent covariate,<br>{iv} all other risk factors as time-fixed (baseline) covariates.	(HR=1.40, p<0.0001). These inferences address the first, fourth and fifth research question.
Step 2:<br><br>A frailty model with the Cox PH framework is fit to model "Time-to-Recurrent Bleeds" with –<br><br>{i} treatment exposure (LAAO vs OAC) as time-dependent covariate.<br>{ii} all other risk factors as time-fixed (baseline) covariates.<br><br>The term "frailty" refers to the different degree of frailness/weakness between different subjects which potentially causes different number of bleeding recurrences for different subjects. Thus the "frailty" is a measure of unobserved heterogeneity between the subjects not accounted for by the usual risk factors.	After adjusting for the effect of other risk factors, LAAO reduces the risk of bleeds compared to OAC but this reduction in risk is not statistically significant (HR=0.83, p=0.2027). Further, a non-zero estimate of the frailty variance parameter shows evidence of unmeasured heterogeneity not explained by other risk factors (point estimate = 1.32, p<0.0001). These inferences address the second research question.
Step 3:<br><br>A Cox PH model for "Time-to-Nonfatal Stroke" is fit with –<br><br>{i} treatment exposure (LAAO vs OAC) as time-dependent covariate.<br>{ii} all other risk factors as time-fixed (baseline) covariates.	There is no evidence to indicate that the risk of non-fatal stroke is different for LAAO vs OAC after adjusting for other risk factors. This inference address the third research question.

© 2025 Dhar G et al. *JAMA Network Open*

Challenges faced:<br><br>The models listed in Step 1, 2 and 3 are "separate" models for Time-to-Death, Time-To-Recurrent Bleeds and Time-To-Nonfatal Stroke respectively. While they do address the five research questions, they do so separately and have one major limitation	Limitation:<br><br>The overall survival benefit seen for LAAO vs OAC (HR=0.71, p=0.0216) is not being explained in a clinically meaningful way – that is, why do we see a survival benefit in "time-to-death" when there is no statistical evidence of reduced risk in bleeding or stroke?
Step 4:<br><br>To address the limitation mentioned above and taking into account results of the models explained in Step 1-3, we fit a more advanced "Joint Frailty Model".<br><br>Specifically, this model aims to address the myriad complexities of our data (See Data Structure #4) while being able to draw inferences on all five research questions in a clinically meaningful way.<br><br>Here, we jointly model "Time-to-Death" and "Time-to-Recurrent Bleeds" while treating treatment exposure and nonfatal stroke as time-dependent covariates. All other risk factors are time-fixed (baseline) covariates. The modeling framework is the usual proportional hazards (PH) framework, but the two outcomes of "death" and "recurrent bleeds" are being modeled jointly. As "recurrent bleeds" indicate a possibility of more than one bleed per subject, joint model also has a frailty component to it.	Main Results:<br><br>After adjusting for the effect of other risk factors, LAAO shows evidence of reduced risk of bleeding compared to OAC (HR=0.70, p=0.0083). The statistically significant non-zero estimate of the frailty variance parameter (point estimate = 0.805, p<0.0001) indicates presence of unmeasured heterogeneity not accounted for by the risk factors included in the model. The point estimate of the "association parameter" is significantly greater than 0 (point estimate = 2.104, p<0.0001) indicating a significant correlation between "recurrent bleeds" and "death" – hence the need to model them jointly. Further, the 95% confidence interval is 1.19-3.02 and as the lower limit is greater than 1, it indicates that when modeling "death" and "bleeds" jointly, "death" acts as a competing event for "bleeds" and that had "death" not occurred (hypothetically speaking),we would have observed more "bleeding events". Lastly, after accounting for the correlation between "death" and "bleeds", the effect of a non-fatal stroke on death is statistically not significant (HR=1.19, p=0.2906).

© 2025 Dhar G et al. *JAMA Network Open*

Modeling challenges faced:	The dataset is quite complex. We have done our best to obtain clinically meaningful results fitting both simpler usual Cox models as well as the more advanced Joint Frailty Model keeping in mind the main research questions.

© 2025 Dhar G et al. *JAMA Network Open*

eFigure 1. Log-log survival plots versus time for categorical covariates in relation to bleeds

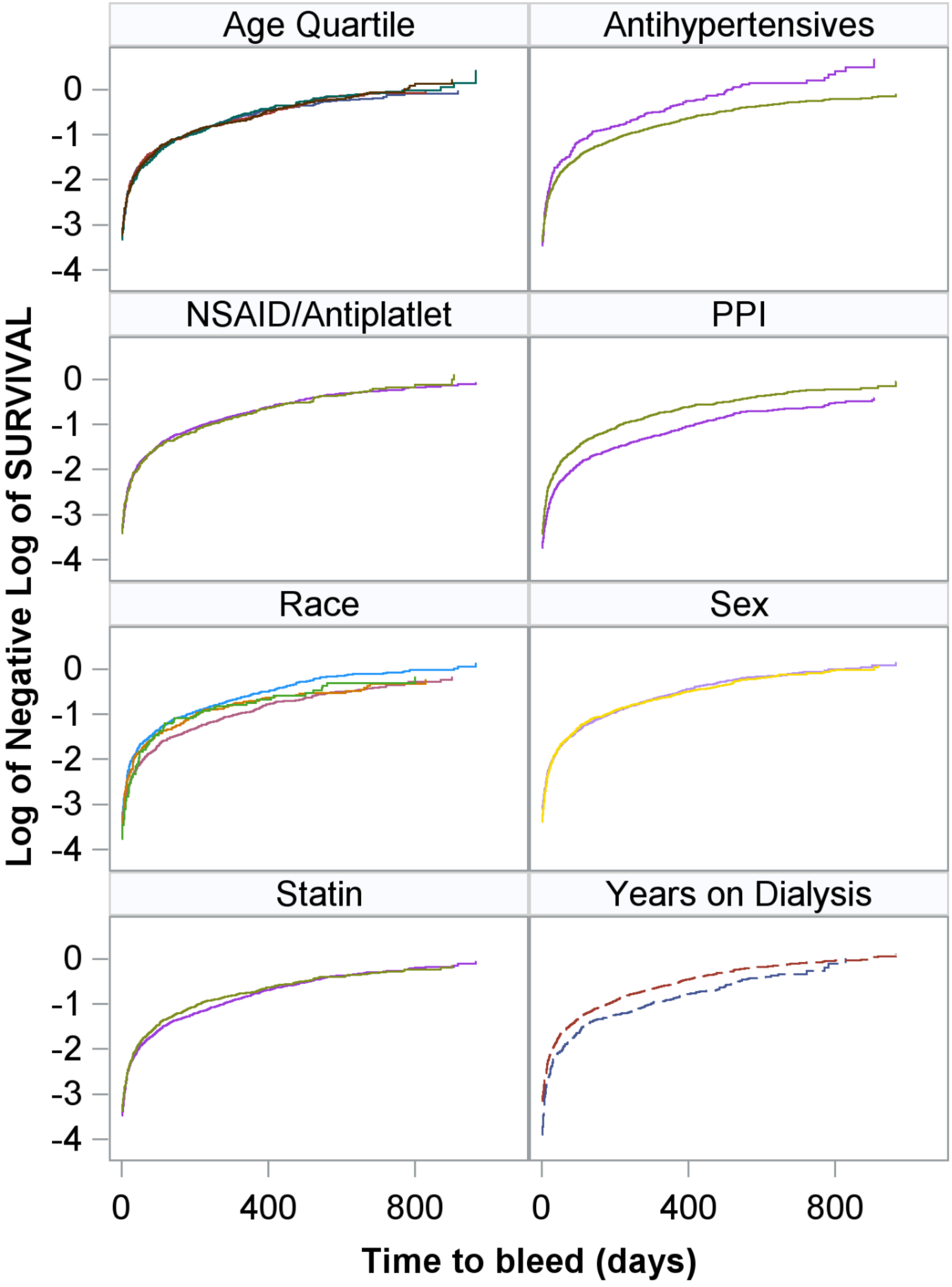

© 2025 Dhar G et al. *JAMA Network Open*

eFigure 2. Log-log survival plots versus time for categorical covariates in relation to death

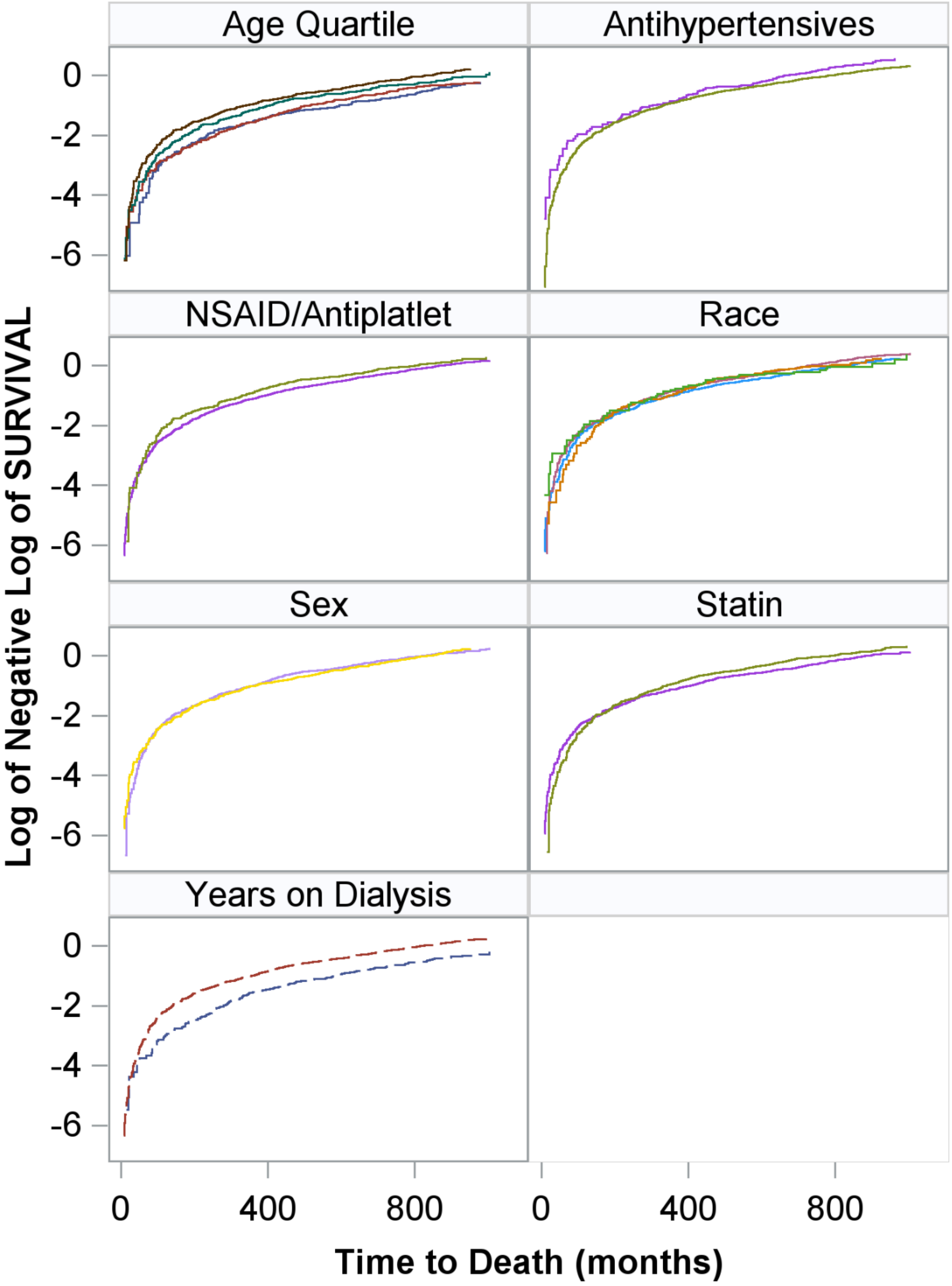

© 2025 Dhar G et al. *JAMA Network Open*

eFigure 3. Plots of Schoenfeld residuals versus time for continuous covariates in relation to bleeding events

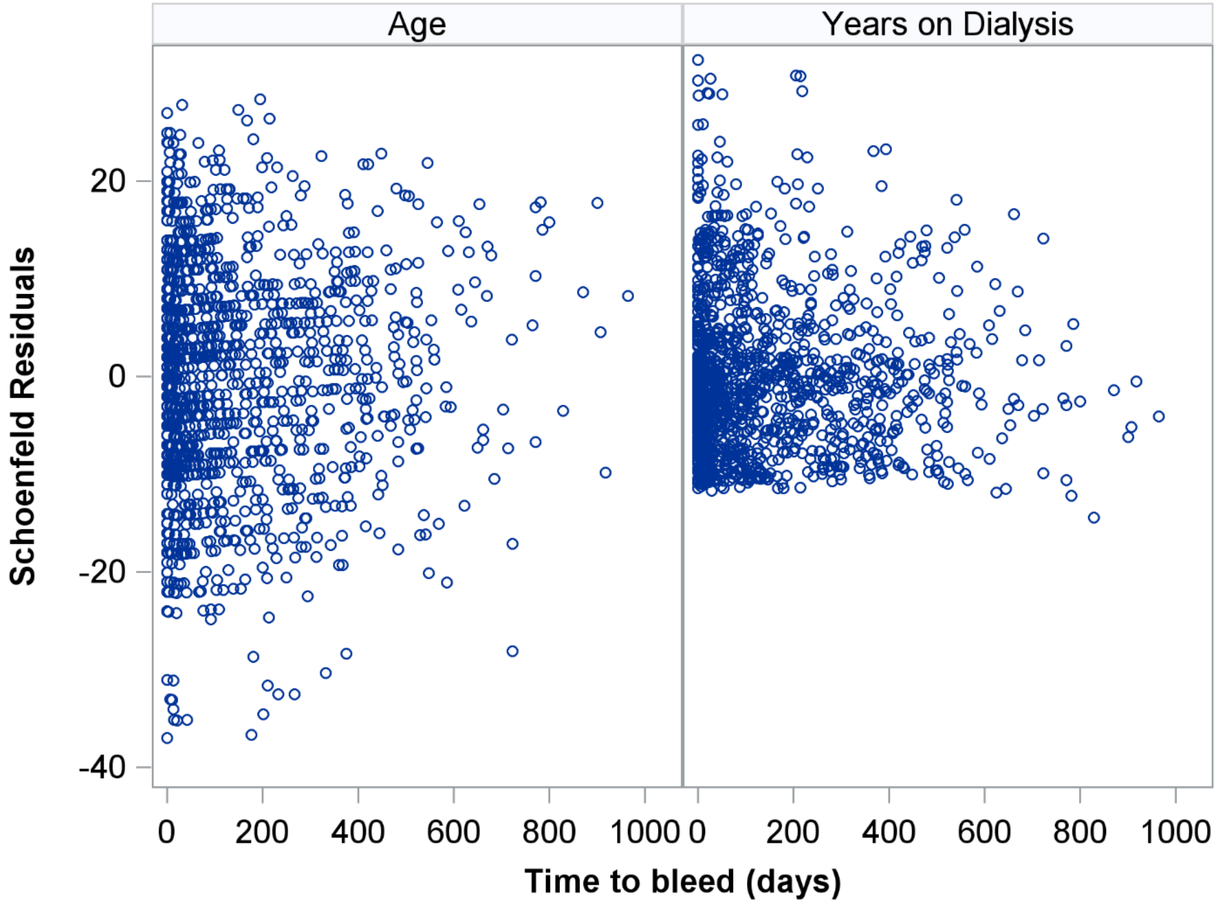

© 2025 Dhar G et al. *JAMA Network Open*

eFigure 4. Plots of Schoenfeld residuals versus time for continuous covariates in relation to death

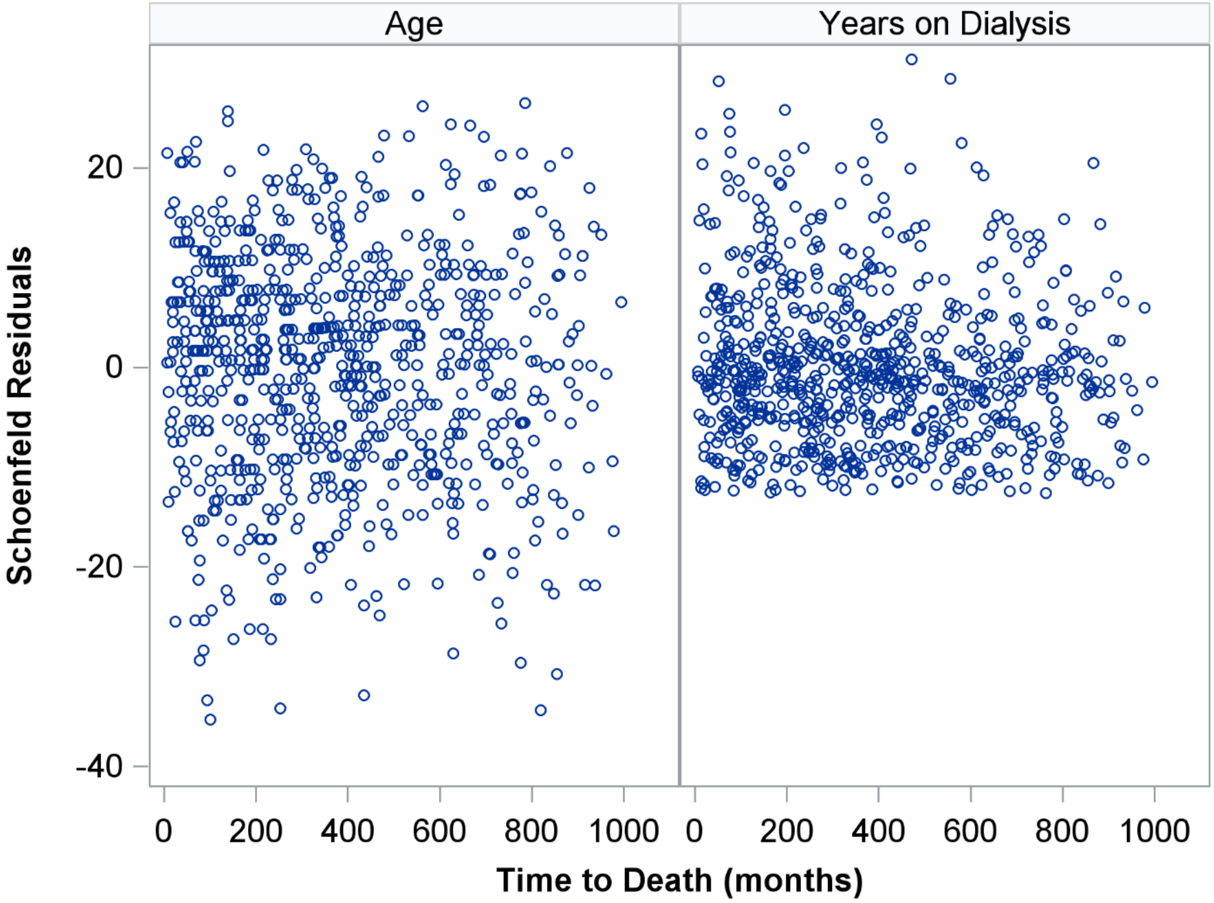

© 2025 Dhar G et al. *JAMA Network Open*
