# Supplementary material for: Left Atrial Appendage Occlusion vs Anticoagulants in Dialysis With Atrial Fibrillation
Source: JAMA Netw Open. 2025 Sep 9;8(9):e2530990. doi: 10.1001/jamanetworkopen.2025.30990 (PMC12421340; doi:10.1001/jamanetworkopen.2025.30990)
Supplement: Supplement 2. — Data Sharing Statement [file jamanetwopen-e2530990-s002.pdf]

Data Sharing Statement

Dhar. Left Atrial Appendage Occlusion vs Anticoagulants in Dialysis With Atrial Fibrillation. *JAMA Netw Open*. Published September 09, 2025. doi:10.1001/jamanetworkopen.2025.30990

Data
Data available: No

Additional Information
Explanation for why data not available: Data is part of the data Use Agreement with USRDS and cannot be shared.
